# Systematic metabolomic studies identified adult adiposity biomarkers with acetylglycine associated with fat loss *in vivo*

**DOI:** 10.3389/fmolb.2023.1166333

**Published:** 2023-04-14

**Authors:** Kuan-Jui Su, Xing-Ying Chen, Rui Gong, Qi Zhao, Shi-Di Hu, Mei-Chen Feng, Ye Li, Xu Lin, Yin-Hua Zhang, Jonathan Greenbaum, Qing Tian, Hui Shen, Hong-Mei Xiao, Jie Shen, Hong-Wen Deng

**Affiliations:** ^1^ Tulane Center for Biomedical Informatics and Genomics, School of Medicine, Tulane University, New Orleans, LA, United States; ^2^ Department of Biostatistics and Data Science, School of Public Health and Tropical Medicine, Tulane University, New Orleans, LA, United States; ^3^ Shunde Hospital of Southern Medical University (The First People’s Hospital of Shunde), Foshan, China; ^4^ Department of Endocrinology and Metabolism, The Third Affiliated Hospital of Southern Medical University, Guangzhou, China; ^5^ Department of Cadre Ward Endocrinology, Gansu Provincial Hospital, Lanzhou, China; ^6^ Department of Preventive Medicine, College of Medicine, University of Tennessee Health Science Center, Memphis, TN, United States; ^7^ Center of System Biology, Data Information and Reproductive Health, School of Basic Medical Science, Central South University, Changsha, China

**Keywords:** obesity, body fat, metabolomics, acetylglycine, biomarkers

## Abstract

Obesity is associated with various adverse health outcomes. Body fat (BF) distribution is recognized as an important factor of negative health consequences of obesity. Although metabolomics studies, mainly focused on body mass index (BMI) and waist circumference, have explored the biological mechanisms involved in the development of obesity, these proxy composite measures are not accurate and cannot reflect BF distribution, and thus may hinder accurate assessment of metabolic alterations and differential risk of metabolic disorders among individuals presenting adiposity differently throughout the body. Thus, the exact relations between metabolites and BF remain to be elucidated. Here, we aim to examine the associations of metabolites and metabolic pathways with BF traits which reflect BF distribution. We performed systematic untargeted serum metabolite profiling and dual-energy X-ray absorptiometry (DXA) whole body fat scan for 517 Chinese women. We jointly analyzed DXA-derived four BF phenotypes to detect cross-phenotype metabolite associations and to prioritize important metabolomic factors. Topology-based pathway analysis was used to identify important BF-related biological processes. Finally, we explored the relationships of the identified BF-related candidate metabolites with BF traits in different sex and ethnicity through two independent cohorts. Acetylglycine, the top distinguished finding, was validated for its obesity resistance effect through *in vivo* studies of various diet-induced obese (DIO) mice. Eighteen metabolites and fourteen pathways were discovered to be associated with BF phenotypes. Six of the metabolites were validated in varying sex and ethnicity. The obesity-resistant effects of acetylglycine were observed to be highly robust and generalizable in both human and DIO mice. These findings demonstrate the importance of metabolites associated with BF distribution patterns and several biological pathways that may contribute to obesity and obesity-related disease etiology, prevention, and intervention. Acetylglycine is highlighted as a potential therapeutic candidate for preventing excessive adiposity in future studies.

## 1 Introduction

Obesity, defined as an excess of adiposity, is a global epidemic widely recognized as a leading cause of various metabolic disorders and cancers (Cirulli et al., 2019; Koenen et al., 2021; He et al., 2023). Excess central fat accumulation causing metabolic disturbance can exacerbate the risk of diabetes and cardiovascular diseases (Vasan et al., 2018; Marsh et al., 2023). Biomarkers reported to influence abdominal obesity have also been shown to be prognostic of early cardiometabolic risk independent of general obesity (Supriya et al., 2018). However, not all abdominal areas in which fat accumulates are associated with harm. It is known that android fat deposited around the waist increases the risk of metabolic disease, while gynoid fat deposited around the hips does not, and some studies suggest gynoid fat may have a beneficial effect (Vasan et al., 2018; Marsh et al., 2023). Therefore, uncovering novel biomarkers for fat distribution can provide more precise insight into fat accumulation and obesity-related diseases.

Metabolomics provides comprehensive profiling of distinct exogenous/endogenous small molecules functioning as intermediates or end products of cellular metabolism. Since metabolites represent the downstream expression of genomic, transcriptomic, and proteomic factors, their study can reveal biomarkers and pathways that link genotypes to phenotypes (Cirulli et al., 2019). Multiple studies have revealed crucial metabolites for obesity or regulating BF (Chen et al., 2015; Bogl et al., 2016; Ahmad et al., 2022; O’Keeffe et al., 2022), some of which were used as a basis for developing treatments and preventive interventions for obesity-related disorders (Okosun et al., 2015; Chen and Gerszten, 2020) and prediction models for visceral adipose tissue, an important prognostic factor that is difficult to measure in practice (Boone et al., 2022). Although there have been numerous efforts to identify metabolic biomarkers of obesity, these studies were largely limited by small sample sizes, the number of metabolites considered, and lack of replication/functional validation. Moreover, they primarily relied on proxy measures of body composition such as BMI and/or waist-to-hip ratio. These proxy measures may miss valuable information provided by the regional BF, and therefore hinder assessment of metabolic alterations and differential risk of metabolic disorders among individuals presenting adiposity differently throughout the body (Rangel-Huerta et al., 2019).

In the present study, we aim to identify shared and specific metabolites and their associated pathways related to various BF traits. The study has three main components: discovery, validation, and *in vivo* experiments. First, we analyzed untargeted liquid chromatography-mass spectrometry (LC-MS) metabolomics profiling on a sample of 517 Chinese women for discovery. Next, we verified the identified BF-related metabolites in two independent cohorts with various ethnicities and genders. Both the validated metabolites and those associated with all the studied BF traits were selected for more detailed examination of their biological functions and relevance as revealed in prior studies. One such metabolite, meeting both criteria, was chosen for *in vivo* experiments for further validation and functional significance in mice. Our study design and analytical approach are outlined in Supplementary Figure S1.

## 2 Materials and methods

### 2.1 Subjects and study design

A total of 517 unrelated peri-/post-menopausal Chinese women in the discovery cohort were recruited from the Third Affiliated Hospital of Southern Medical University (Guang Zhou, Guang Dong Province, China) between June 2014 and January 2018. The mean age was 52.9 years (standard deviation (SD) = 2.9). Two independent samples for validation were sampled from the Louisiana Osteoporosis Study (LOS) (Zhao et al., 2018). The first validation cohort consisted of 136 Caucasian women (mean aged 31.48 years (SD = 5.08)) sampled by a discordant phenotype design based on the top/bottom 20% of the hip bone mineral density Z-score (Zhao et al., 2018). The second validation cohort comprised 700 males (295 African Americans and 405 Caucasians) with mean age 37.8 (SD = 8.45) years overall. The study designs and sample characteristics are detailed in Supporting Material S1 and Supplementary Tables S1-S2, respectively. Signed consent forms were obtained from all participants. All clinical measurements and experiment procedures were subject to the Helsinki Declaration II and regulations of the Institutional Review Boards.

### 2.2 Clinical measurements

All respondents in the discovery and validation cohorts completed a questionnaire regarding demographics, lifestyle, dietary factors, and reproductive and medical history. Trained research staff collected clinical measurements with participants. The BF distribution phenotypes considered in this study were android fat/whole BF mass ratio (A/W ratio), gynoid fat/whole BF mass ratio (G/W ratio), android fat to gynoid fat ratio (A/G ratio), and whole BF percentage (W%) (Bogl et al., 2016). Daily calibrated DXA machines (discovery cohort: Lunar, GE Healthcare, Madison, WI, United States; validation cohorts: Hologic Inc., Bedford, MA, United States) were used to measure BF (Supplementary Figure S2). The detail of the region of interest is provided in Supporting Material S1.

### 2.3 Metabolite analysis

Untargeted LC-MS metabolite profiling was conducted on serum samples from all cohorts. The details of experimental protocols, which are in accordance with the suggestions of the Metabolomics Standards Initiative reporting standards (Sumner et al., 2007), have been previously described (Gong et al., 2021) and are provided in Supporting Material S2. A high-resolution tandem mass spectrometer TripleTOF5600plus (SCIEX, United Kingdom) at Lian-Chuan Biotechnology Co., Ltd (Hangzhou, China) was used to detect metabolites eluted from the column in the discovery cohort. LC-MS metabolomics platforms were also used to perform the metabolomic analyses on serum samples for both validation cohorts. The experimental protocols for the validation cohorts have been previously described (Zhao et al., 2018) and are listed in Supporting Material S2. All metabolites profiling with a unique or high-confidence annotation were log-transformed and auto-scaled prior to the subsequent analyses.

### 2.4 *In vivo* validation study in mice for acetylglycine

Acetylglycine consistently demonstrated an anti-obesity association with observed BF traits across sex and ethnicity. We performed an *in vivo* study that extended the work of Harper et al. (Harper et al., 2010) by exploring the effect of acetylglycine on high-fat diet-induced obese mice for further validation. A total of 61 female C57BL/6 J mice were randomized into five groups: 1) standard chow diet with vehicle (control; *n* = 12), 2) high fat (60% kcal from fat; *n* = 12) diet with vehicle (HFD; *n* = 12), 3) HFD with low-dosage acetylglycine (HFD + ACE500; 500 mg/kg; *n* = 12), 4) HFD with medium-dosage acetylglycine (HFD + ACE1000; 1,000 mg/kg; *n* = 12), and 5) HFD with high-dosage acetylglycine (HFD + ACE1500; 1,500 mg/kg; *n* = 13). We administered drinking water or acetylglycine (treatment) to corresponding mice by oral gavage every day starting at 8 weeks of age. Experimental measurements were recorded weekly during the 16-week intervention. We used micro-computed tomography (µ-CT) to measure total abdominal fat (between L1 and L5 vertebrae), visceral BF, and subcutaneous BF (Luu et al., 2009). The biochemical analysis involved the measurement of fasting plasma glucose (FPG) after 6-h and 12-h fasting, total cholesterol (TC), triglyceride (TG), high-density lipoprotein cholesterol (HDL), low-density lipoprotein cholesterol (LDL), alanine transaminase (ALT), and aspartate aminotransferase (AST). All experimental procedures are provided in Supporting Material S3.

### 2.5 Statistical analysis

We investigated the relationship between the metabolites and BF traits using two complementary approaches, seemingly unrelated regression (SUR) and sparse partial least squares (sPLS) regression. The most important BF-related metabolites were identified as those with variable importance in projection (VIP) score >1 in sPLS and SUR false discovery rate (FDR) *q*-value ≤0.2. The selection of cutoffs for the FDR *q*-value and VIP score usually depends on the study objective. In this study, we aimed to provide a robust list of potential BF-related metabolites for future replication, and therefore chose not to apply highly stringent significance criteria since the analysis is exploratory in nature. A pathway analysis was performed to identify biological functions with *p*-score <0.05. The Spearman and partial Spearman correlation tests were conducted in the validation cohorts to examine the associations between the metabolites and traits after adjustment for age, height, physical activity for both cohorts, and ethnicity in the second cohort. We also performed the tests stratified by ethnicity. *p*-value ≤0.05 was considered statistically significant. All analyses were detailed in Supporting Material S4. The metabolites associated with all BF traits in the discovery cohort or replicated the associations in the validation cohorts will be further discussed in our study (Supporting Material S5).

The Generalized Estimating Equations (GEE) model was applied to investigate the associations between acetylglycine and the measure of weight and weight gain in the animal models. The covariates included baseline weight, treatment (water, low-, medium-, or high-dose acetylglycine), and diet (normal/HFD) in the weight model, and treatment and diet in the weight gain model. Tukey method was used to examine whether there were significant pairwise differences (*p*-value ≤0.05) in weight and weight gain. The Kruskal–Wallis test was used as a global test for a one-time measurement to compare biomedical indexes across different treatment groups. The null hypothesis of this test is that there is no significant difference in the biomedical measurement across all treatment groups, while the alternative hypothesis is that at least one group differs significantly from at least one other group. Following the Kruskal–Wallis test, Wilcoxon tests were conducted to examine whether acetylglycine had significant differences (FDR q-value ≤0.05) in pairwise comparisons for both the µ-CT and biochemical measurements.

## 3 Results

### 3.1 Relationships between BF traits and serum metabolites in discovery

**FIGURE 1:**
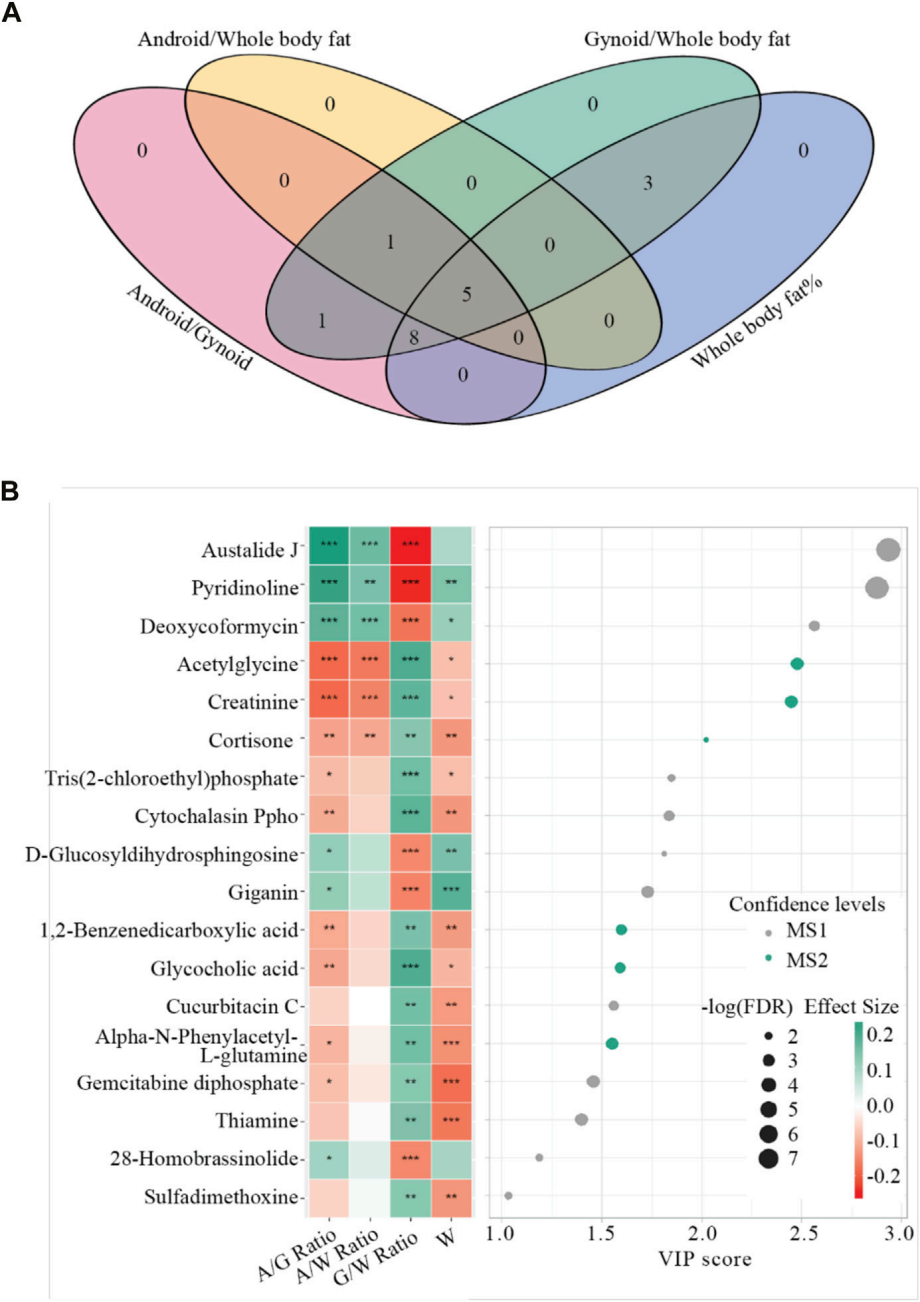
Summary results for the joint analysis in the discovery cohort. Panel **(A)** Venn diagram depicting metabolite sets that were significantly associated with body fat traits (FDR q-value <0.2 and VIP >1). Traits are represented by ovals of different colors. The number in each area represents the count of identified BF-associated metabolites associated with the given traits. Panel **(B)**. Important metabolites selected on the basis of VIP, score >1 and FDR q-value ≤0.2. Each tile represents the effect size of the metabolite on each trait. VIP: the variable importance in projection scores, FDR: false discovery rate, A/W ratio: Android fat/Whole body fat ratio, G/W ratio: Gynoid fat/Whole body fat ratio, A/G ratio: Android fat to gynoid fat ratio, and W: Whole body fat %. *: *p*-value <0.05; **: *p*-value <0.01; ***: *p*-value <0.001.

Among 3,075 annotated metabolites with MS1 and MS2 confidence levels in our discovery cohort (Supplementary Table S3 and Supporting Material S3), we identified 18 statistically significant BF-metabolite associations with FDR *q*-values <0.2 in the SUR models and VIP scores >1 in sPLS (Figure 1 and Supplementary Table S4). Figure 1A illustrates how the metabolites relate to multiple traits: none of the 18 metabolites exclusively related to a single BF trait. All were significantly associated with G/W ratio. Overall, two metabolites had significant relationships with three traits (G/W, A/W, and A/G ratios) representing central BF and five with all four traits representing general and central BF.

Directions of coefficient effects on the four traits were consistent across the 18 metabolites; A/G ratio, A/W ratio, and W% were always affected in opposite directions as G/W ratio (Figure 1B). Those having positive relationships with A/G ratio, A/W ratio, and W% but a negative relation with G/W ratio were defined as obesity-risk metabolites, the others as protective metabolites. Among the six metabolites with VIP scores >2, the top three (austalide J, pyridinoline, and deoxycoformycin) are obesity-risk metabolites, while acetylglycine, creatinine, and cortisone are protective metabolites. The top six in the VIP score also had relatively large effect sizes on central BF compared with general BF (Supplementary Figure S3). Acetylglycine, for example, had stronger negative effect sizes on A/G ratio (*β* = −0.19, *p*-value = 1.09 × 10^−5^) and A/W ratio (*β* = −0.17, *p*-value = 6.23 × 10^−5^) than on W% (*β* = −0.09, *p*-value = 3.24 × 10^−3^).

### 3.2 Functional topological pathway analysis in discovery

Six metabolites out of 18 could be mapped to the Kyoto Encyclopedia of Genes and Genomes (KEGG) database and used in the pathway analysis. We identified 14 significant pathways, three modules, 28 enzymes, 39 reactions, and 21 compounds (refer to Table 1 for an overview and Supplementary Table S6 for a comprehensive list). The topological results showed that thiamine had the highest connectivity at the compound level (degree = 10) and thiamine metabolism had the highest connectivity at the pathway level (degree = 9) in Supplementary Figure S4. The connectivity degree indicates the importance of thiamine for essential metabolic processes.

**TABLE 1:** Summary of topological attributes of the top 5% significant biological components at each biological level.

Level	KEGG ID	KEGG name	p Score	Betweenness centrality (B)	Closeness centrality (C)	Combined score (B)+(C)	Degree
Pathway^a^	hsa00330	Arginine and proline metabolism	1.80 × 10^−3^	0.35	0.54	0.89	5
	hsa02010	ABC transporters	1.59 × 10^−4^	0.39	0.26	0.64	8
Module^a^	hsa04979	Cholesterol metabolism	1.00 × 10^−6^	0.31	0.25	0.56	5
	M00047	Creatine pathway	1.00 × 10^−6^	0.37	0.56	0.93	5
Enzyme^b^	2.3.2.27	RING-type E3 ubiquitin transferase	1.54 × 10^−3^	0.38	0.24	0.62	4
	2.7.3.2	Creatine kinase	1.00 × 10^−6^	0.04	0.42	0.46	2
	2.1.1.2	Guanidinoacetate N-methyltransferase	1.00 × 10^−6^	0.05	0.41	0.45	2
	3.1.3.2	Acid phosphatase	8.83 × 10^−5^	0.15	0.23	0.37	4
	2.1.4.1	Glycine amidinotransferase	1.84 × 10^−3^	0.00	0.37	0.37	1
	3.1.1.3	Triacylglycerol lipase	1.96 × 10^−2^	0.08	0.24	0.32	2
Reaction^c^	R07420	Phosphocreatine ≤≥ Creatinine + Orthophosphate	1.00 × 10^−6^	0.13	0.47	0.60	3
	R01884	Creatinine amidohydrolase	1.00 × 10^−6^	0.10	0.47	0.57	3
	R02922	Creatinine iminohydrolase	1.00 × 10^−6^	0.09	0.42	0.51	3
	R01566	Creatine amidinohydrolase	9.33 × 10^−4^	0.04	0.43	0.47	2
	R01881	ATP:creatine N-phosphotransferase	2.01 × 10^−6^	0.08	0.39	0.47	3
	R03720	Choloyl-CoA:glycine N-choloyltransferase	4.33 × 10^−3^	0.18	0.25	0.44	8
	R03187	N-Methylimidazolidine-2,4-dione amidohydrolase	3.68 × 10^−2^	0.04	0.38	0.42	2
Compound^d^	C00300	Creatine	9.93 × 10^−5^	0.17	0.44	0.61	4
	**C00378**	**Thiamine**	**1.00 × 10** ^**−6**^	**0.27**	**0.26**	**0.53**	**10**
	**C00791**	**Creatinine**	**1.00** × 10^−6^	**0.08**	**0.44**	**0.52**	**3**
	C00037	Glycine	1.16 × 10^−5^	0.22	0.26	0.48	5
	**C01921**	**Glycocholate**	**1.00 × 10** ^**−6**^	**0.21**	**0.24**	**0.46**	**3**
	C02305	Phosphocreatine	1.35 × 10^−4^	0.02	0.38	0.41	2
	**C01606**	**Phthalate (1,2-Benzenedicarboxylic acid)**	**1.00 × 10** ^**−6**^	**0.09**	**0.21**	**0.30**	**4**
	**C00762**	**Cortisone**	**1.00 × 10** ^**−6**^	**0.11**	**0.18**	**0.29**	**6**
	**C04148**	**Phenylacetylglutamine (Alpha-N-phenylacetyl-L-glutamine)**	**1.00 × 10** ^**−6**^	**0.00**	**0.18**	**0.18**	**1**

^a^ Reported pathways and modules with combined score (B + C) > 0.5.

^b^ Reported enzymes with combined score >0.3.

^c^ Reported reactions with combined score >0.4.

^d^ Reported compounds with combined score >0.4 or original input compounds; **Bold,** the original BF-related metabolites. The components were selected by the threshold of combined score greater than 0.3 or as the input metabolites. A comprehensive list can be found in Supplementary Table S6.

### 3.3 Metabolite and BF trait relations validation in LOS cohorts

We examined the correlations of four metabolites in the female validation cohort and five in the male validation cohort, which resulted in 22 significant relations between the metabolites and traits (Table 2). The correlations between acetylglycine and the traits were significant in both cohorts, with the strongest negative association with W% and A/W ratio respectively in Caucasian women (Spearman correlation *ρ*) = −0.34, partial Spearman correlation (ρ′) = −0.29, both *p*-value < 0.001) and African American and Caucasian men (*ρ* = −0.28, ρ' = −0.26, both *p*-value < 0.001). Glycocholic acid was negatively associated with A/G ratio, A/W ratio, and W% in African American and Caucasian men only, which may be attributed to the relatively small sample size of Caucasian women (Supplementary Tables S7-S14).

**TABLE 2:** Summary of body-fat associated metabolites in the validation cohorts.

Metabolite	Class	Ion	m/z	RT/RI^a^	Molecular	Trait	ρ	ρ *p*-value	ρ′	ρ' *p*-value
Validation 1: Caucasian women (*n* = 136)										
Acetylglycine	Amino acids	Pos	140.03	1.17	C_4_H_7_NO_3_	AG	−0.31	1.96 × 10^−4^	−0.28	9.10 × 10^−4^
						AW	−0.29	6.89 × 10^−4^	−0.25	3.27 × 10^−3^
						GW	0.29	5.88 × 10^−4^	0.28	1.17 × 10^−3^
						W%	−0.34	5.17 × 10^−5^	−0.29	6.55 × 10^−4^
Creatinine	Amino acids	Neg	112.05	0.98	C_4_H_7_N_3_O	AW	−0.20	1.96 × 10^−2^	−0.15	7.61 × 10^−2^
Thiamine	Pyrimidines	Pos	265.11	1.13	C12H_16_N_4_OS	W%	−0.19	2.34 × 10^−2^	−0.16	7.01 × 10^−2^
Validation 2: African American and Caucasian men (n = 700)										
Acetylglycine	Amino acids	Polar	116.04	1,780	C_4_H_7_NO_3_	AG	−0.26	7.22 × 10^−12^	−0.24	1.47 × 10^−10^
						AW	−0.28	6.48 × 10^−14^	−0.26	1.97 × 10^−12^
						GW	0.09	1.41 × 10^−2^	0.09	1.32 × 10^−2^
						W%	−0.27	6.34 × 10^−13^	−0.24	5.28 × 10^−11^
Cortisone	Lipid	Neg	359.19	4,575	C_21_H_28_O_5_	AG	−0.11	2.67 × 10^−3^	−0.10	1.12 × 10^−2^
						AW	−0.11	2.38 × 10^−3^	−0.10	8.97 × 10^−2^
						W%	−0.11	5.02 × 10^−3^	−0.09	1.22 × 10^−2^
Creatinine	Amino acids	Pos Early	114.07	2,055	C_4_H_7_N_3_O	AG	−0.09	2.20 × 10^−2^	−0.05	ns
						AW	−0.09	1.84 × 10^−2^	−0.03	ns
						W%	−0.16	1.24 × 10^−5^	−0.13	6.69 × 10^−4^
Glycocholic acid	Lipid	Neg	464.30	5,163	C_26_H_43_NO_6_	AG	−0.10	8.16 × 10^−3^	−0.07	ns
						AW	−0.13	5.96 × 10^−4^	−0.10	8.13 × 10^−3^
						W%	−0.12	1.96 × 10^−3^	−0.09	1.38 × 10^−2^
N-Phenylacetyl-L-Glutamine	Peptide	Pos Early	265.12	2,145	C_13_H_16_N_2_O_4_	AG	−0.02	ns	−0.11	4.17 × 10^−3^
						AW	−0.04	ns	−0.12	1.34 × 10^−3^
						W%	−0.08	2.81 × 10^−2^	−0.14	2.60 × 10^−4^

m/z: mass-to-chare ratio; a: RT (Retention time) and RI (Retention index); *ρ*: Spearman correlation coefficient; ρ': Partial Spearman correlation coefficient adjusted by age, height, and physical activity in Validation 1 and additional ethnicity in Validation 2; ns: not statistically significant; The directions of the correlation test in the validation studies are consistent with the findings in the Chinese women cohort. A/G: android to gynoid ratio; A/W: android fat to whole body total fat; G/W: gynoid fat to whole body total fat; W%: whole body total fat percentage.

In the ethnicity-stratified analyses, we replicated the correlations of five metabolites related to at least one BF trait in African American or Caucasian men (Supplementary Table S5). Acetylglycine had the highest absolute correlation coefficients on the BF traits (except for the G/W ratio in the African American men). Cortisone had the significant negative correlations with A/G ratio, A/W ratio, and W% exclusively in the African American men; creatinine was also negatively related to W% in Caucasian men.

### 3.4 Relationship of acetylglycine with body weight, body weight gain, and abdominal fat mass in mice

The acetylglycine treatments with equivalent baseline weight and diet were significantly associated with an average decrease in body weight of 0.51 (*p*-value = 0.01) and 1.03 (*p*-value = 2.30 × 10^−7^) grams, respectively, in ACE1000 and ACE1500 groups, compared with groups without the treatment. Similarly, we found negative marginal effect sizes on weight gain while adjusting for diet (Table 3). Body weight and weight gain were lower in all the acetylglycine treatment groups and the control group than the HFD-only group (Supplementary Figure S5). Overall food intake presented no differences between the HFD-only and HFD with treatment groups during the intervention period.

**TABLE 3:** Results of GEE models for the associations of acetylglycine on mouse weight and weight change.

Variable	Effect size	SE	Wald	*p*-value
*Trait: Weight*				
Water	REF			
TRT ACE 500	−0.29	0.28	1.08	0.30
TRT ACE 1000	−0.51	0.20	6.56	0.01
TRT ACE 1500	−1.03	0.20	26.78	2.30 × 10^−7^
Normal Diet	REF			
HFD	1.35	0.19	48.23	3.80 × 10^−12^
Baseline weight	0.98	0.10	92.78	<2.00 × 10^−16^
*Trait: Weight change*				
Water	REF			
TRT ACE 500	−0.38	0.32	1.44	0.2295
TRT ACE 1000	−0.65	0.22	8.61	0.0033
TRT ACE 1500	−1.41	0.23	39.10	3.90 × 10^−10^
Normal Diet	REF			
HFD	1.61	0.21	58.00	2.70 × 10^−14^

REF: Reference group. HFD: High fat diet. TRT ACE: Treatment group with acetylglycine (dose); SE: Standard error for the effect size.

**FIGURE 2:**
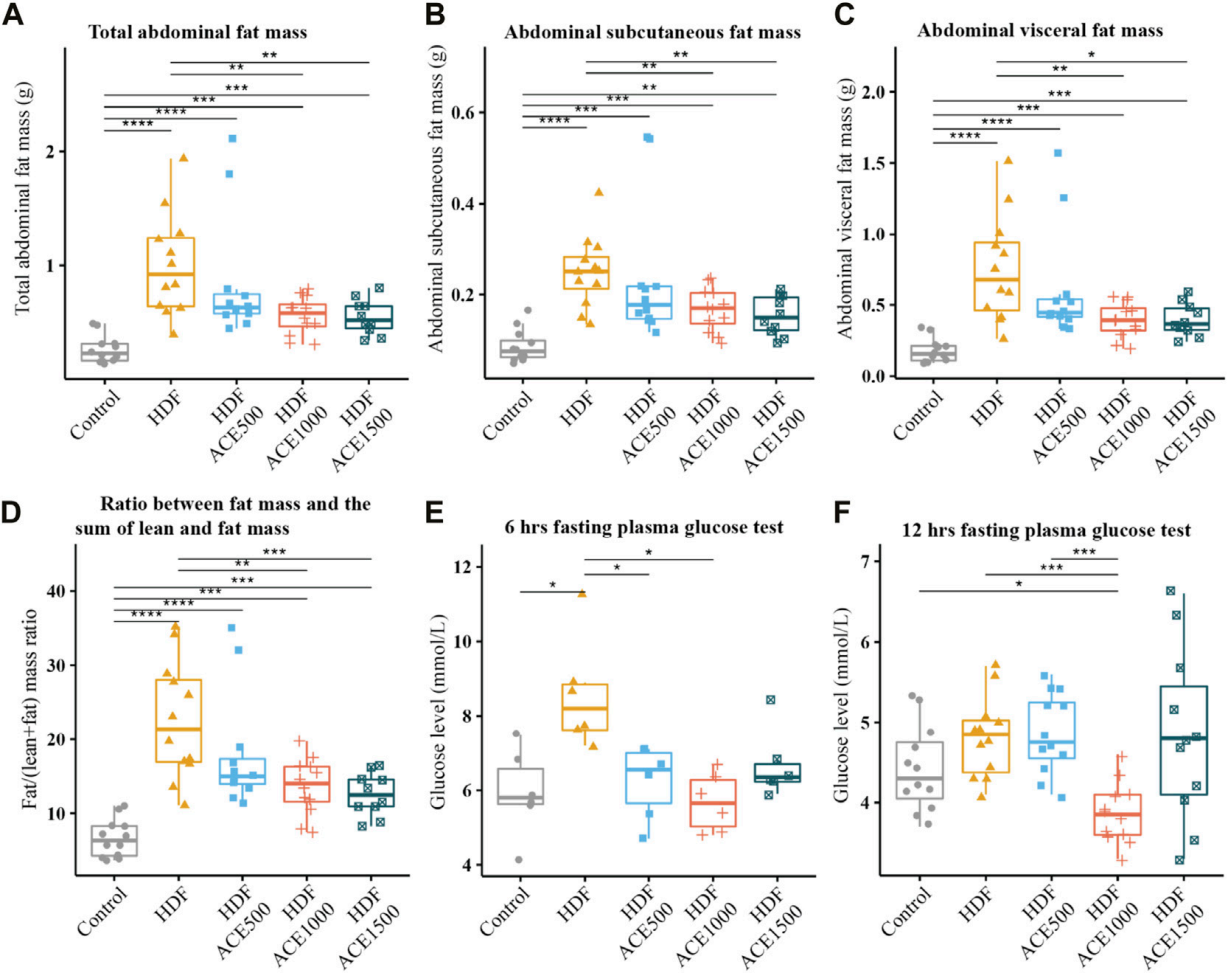
*In vivo* study with acetylglycine. Panels **(A–D)** show the pairwise comparisons for the µ-CT measures among different groups. The abdominal region was defined as between L1–L5 lumbar vertebrae. Panels (**E, F)** are the pairwise comparisons for the fasting plasma glucose test. FDR: false discovery rate; *: FDR *q*-value <0.05; **: FDR *q*-value <0.01; ***: FDR *q*-value <0.001; ****: FDR *q*-value <0.0001.

Among the acetylglycine treatment groups, we observed significant differences in the µ-CT measures: abdominal fat, visceral fat, subcutaneous fat, and fat/weight ratio (Figure 2 and Supplementary Tables S1-S15). The HFD groups had significantly higher measurements than the control group, indicating the success of the obesity model. Despite similar HFD consumption, we observed a decreasing trend in the four µ-CT measures as the treatment dosage increased, and statistically significant differences between the HFD-only group and the HFD with the medium and high-dose groups.

### 3.5 Association of acetylglycine with biochemical measurements in mice

The overall tests indicated significant differences in the levels of glucose, CHO, HDL, and LDL among the groups (Supplementary Table S16). The HFD-only group in the 6-h FPG test had significantly higher glucose level (mean = 8.57 mmol/L, SD = 1.49) than the control, HFD + ACE500, and HFD + ACE1000 groups (Figure 2). In the 12-h FPG test, there were significant differences between the HFD + ACE1000 and control/HFD-only groups. ALT and AST, two enzymes for testing liver toxicity or damage, were not significantly different across groups (Supplementary Figure S6). Lipid profiles were significantly higher in the treatment groups with HFD compared to those in the control group, and there were no differences in the cholesterol levels among the intervention groups (Supplementary Figure S7). Taken together, the HFD-only diet successfully induced obesity in the mice, as reflected by the higher weight, weight gain, abdominal fat mass, and glucose level observed in the study.

## 4 Discussion

We performed a joint analysis that revealed the relationships between the metabolites and four DXA-derived BF measures among Chinese women. In contrast to proxy phenotypes, such as BMI, BF measures provide a direct assessment of body adiposity distribution, enabling the identification of novel metabolites related to general or central BF. We identified 18 metabolites that demonstrated consistent associations with A/G ratio, A/W ratio, and W%, and opposing associations with G/W ratio. We further partially validated the consistent relationships of the metabolites on the BF traits in two independent samples with different ethnicity and sex characteristics. Lastly, we validated the protective effect of acetylglycine in the obese mouse experiments.

Pyridinoline, deoxycoformycin, acetylglycine, creatinine, and cortisone were robustly associated with general and central BF. Austalide J and 28-homobrassinolide were only associated with central BF, and austalide J had the highest VIP score, reflecting a strong contribution in distinguishing abdominal obesity. Interestingly, no metabolite was exclusively related to any single BF trait, implying that the identified metabolites simultaneously influence total and central region fat deposits with different effect sizes. As gynoid fat may reduce the risk of metabolic diseases (Fu et al., 2013; Okosun et al., 2015), our findings of 12 metabolites positively associated with G/W ratio suggest further replication and merit for future study in preventing obesity.

Pyridinoline and deoxycoformycin were observed as positive associations with general and central BF, except gynoid fat, in our study. These two metabolites have been recognized from exogenous sources and have close relationships with complex diseases. Pyridinoline, a well-known biomarker of bone resorption and formation (Delmas et al., 1991; Kuo and Chen, 2017), has been reported to be positively associated with weight, BMI, and bone mineral density at different skeletal sites (New et al., 2000; Wang et al., 2016). It is a cross-linked compound that provides chemical stabilization in cartilage, bone, ligaments, and blood vessels (Kuo and Chen, 2017). Next, deoxycoformycin is an inhibitor of adenosine deaminase and a common anticancer chemotherapeutic drug for leukemia and lymphoma treatment (Tusup et al., 2022). In addition, deoxycoformycin can be produced by fungi such as *C. militaris*, which is regarded as a beneficial food supplement (Chen et al., 2020). However, it increases the susceptibility to toxic accumulation in kidney, liver, and central nervous system (Demain and Sanchez, 2009; Chen et al., 2020). The effect of the impaired tissue/organs by deoxycoformycin on the development of BF and its dose-dependent toxicity in humans is still unclear. Further research on the underlying mechanism of this relationship is warranted.

We demonstrated the potential anti-obesity effect of acetylglycine on both general and central body fat, which is consistent with previous findings of its association with lower BMI levels and fat percentages in population-based studies (Moore et al., 2014; Cirulli et al., 2019). However, a longitudinal study of Mexican American women aged 20–72 years reported that increased levels of acetylglycine elevated the risk of weight gain (Zhao et al., 2016). Further investigations into the long-term effect are needed to clarify these contradictory results. In addition to obesity-related traits, a study on the causal effect of metabolites on cardiovascular diseases found that acetylglycine is associated with a lower risk of diastolic blood pressure and is considered a potential cause (Qiao et al., 2021). The glycine N-acyltransferase (*GLYAT*) enzyme, linked with phenylalanine metabolism (Supplementary Figure S4), forms N-acylglycines (Westhuizen et al., 2000) and catalyzes glycine conjugation of acyl-Coa-species, producing intermediate products such as acetylglycine in amino acid and fatty acid metabolism (Badenhorst et al., 2013). A mice study showed that lower mRNA expression levels of GLYAT in adipose tissue were observed in a fat-susceptible group compared to a fat-resistant group (Fedry et al., 2016). The perturbation of *GLYAT* induces harmful disruption to CoA homeostasis (Badenhorst et al., 2013), musculoskeletal development (Badenhorst et al., 2013), and obesity-related metabolic disturbances (Fedry et al., 2016; Alves et al., 2019), suggesting that acetylglycine plays a role in the development of obesity-related diseases.

Creatinine, a breakdown product of creatine, reflects muscle metabolism and kidney function and plays a significant role in muscle energy production (Patel et al., 2013). Individuals with low muscle mass and body weight have low creatinine levels (Patel et al., 2013) and high glomerular hyperfiltration rates, which may increase the risk of metabolic diseases and diabetes (Hjelmesæth et al., 2010). The association between cortisone and general/central BF was consistent with a recent study reporting a negative association on BMI in European women (Cirulli et al., 2019). Interestingly, when assessing the relationship of cortisone in childhood obesity, cortisol and cortisone levels were significantly positively correlated with BMI and A/G ratio (Noppe et al., 2016).

The age-dependent differences in activities of 11β-hydroxysteroid dehydrogenases, an enzyme catalyzing the interconversion between cortisol and cortisone, may reverse the associations on BF deposition (Vierhapper et al., 2007; Chapman and Seckl, 2008). Cortisone participating in cortisol metabolism for generating steroid hormone is a known signature for obesity, type 2 diabetes (Gawlik et al., 2020), and other diseases (Supplementary Figure S4).

In our validation analyses, we observed the negative relationships for glycocholic acid, a key a bile acid regulator of fat absorption, cholesterol level, and energy homeostasis (Marco-Ramell et al., 2018). Glycocholic acid was only replicated in African American or Caucasian men but not in Caucasian women. This discrepancy could be attributed to aging-related decline in physiological functions, such as a decrease in female estrogen and hormone levels. A study reported a large difference in glycocholic acid levels between obese and lean women aged between 50 and 70 years old, and a relatively small difference in women aged 30–40 years old (Xie et al., 2015). This is consistent with the findings from our study samples. Further work is needed to understand the complex relationship between glycocholic acid and BF which may be mediated by age-related factors, estrogen, and hormone level.

The negative associations for N-Phenylacetyl-L-Glutamine and thiamine were validated in the men’s and women’s replication samples, respectively. A recent study reported that there are no sex differences in excretion of the amino acid N-Phenylacetyl-L-Glutamine (Zheng et al., 2014). The abnormal activity of N-Phenylacetyl-L-Glutamine could be attributed to the difference in phenylalanine metabolism and further reflect the development of obesity (Bogl et al., 2016). However, some studies also observed that the concentration was lower in obese men compared with normal-weight men (Yu et al., 2018). The mechanism through which N-Phenylacetyl-L-Glutamine may influence BF requires further investigation. Moreover, many studies support thiamine as an essential micronutrient in glucose metabolism that is negatively associated with obesity (Maguire et al., 2018). To the best of our knowledge, our study provided the first evidence of the relationship between thiamine and BF.

The arginine and proline metabolism pathway had a high closeness and betweenness centralities, which implies that the pathway has considerable influence on neighboring molecules/pathways. The pathway is known to be associated with obesity (Cirulli et al., 2019). Elevated arginine levels can reduce BF accretion in humans and animals (Wu et al., 2009). Further investigation of the identified biological components involved in the arginine and proline metabolism may reveal an important influence on obesity.

The ATP-binding cassette (ABC) transporter pathway, a pivotal connector pathway, regulates the import/export of membrane proteins, such as thiamine, phthalate, L-glutamine, and glycine *via* different enzymes or reactions for the overall BF-related network (Rees et al., 2009). The pathway may delineate associations between genes and physiologic changes before, during, and after the onset of obesity in mice (Donepudi et al., 2016) and beneficial influences on obese women (Teixeira et al., 2020). However, we observed that phthalate in ABC transporters was negatively associated with A/G, A/W, and W%, in accordance with the findings from Hatch et al.’s study of BMI and waist circumference (Hatch et al., 2008). Other studies found contradictory results, demonstrating that phthalate was an endocrine-disrupting chemical (Apau et al., 2020) and linked to adverse health outcomes such as obesity and diabetes (Hatch et al., 2008; Apau et al., 2020). The various characteristic of phthalates on obesity may depend upon endogenous hormone levels and vary across sex and age groups (Hatch et al., 2008). The influence on BF and other pathways *via* the ABC transporter pathway may warrant further investigation.

Additionally, our study suggested that impaired cholesterol metabolism may cause adverse health outcomes. The cholesterol metabolism pathway was connected to pathways for aldosterone-regulated sodium reabsorption and prostate cancer. Cortisone and cortisol were closely connected to the disease pathway and may be diagnostic biomarkers for obesity. The impaired conversion of cortisone and cortisol has been reported as an important factor for insulin sensitivity and central obesity (Weaver et al., 1998).

Harper et al. observed no statistical differences in body weights and feed consumption between the acetylglycine treatment groups and the control group (Harper et al., 2010). Our animal model extended the prior research by exploring the effect of acetylglycine on HFD-induced obese mice. For the first time, we demonstrated a protective effect of mid and high doses of acetylglycine on body weight and fat in mice. The evidence has also been seen in a case of smoking-cessation-induced weight gain mice study that showed acetylglycine ameliorated weight gain rate compared with HFD control mice (Fluhr et al., 2021). The single-cell transcriptomics analysis of epididymal-adipose immune cells exemplified that acetylglycine is a potent signaling molecule that modulates modulated multiple adipose-tissue gene expressions in obesity-associated pathways such as immune response, lysosome function, and tissue remodeling (Fluhr et al., 2021).

Biochemical tests showed significant increases in the fasting glucose, total cholesterol, HDL, and LDL of the HFD groups, and unaltered triglycerides. This is consistent with what was reported in a previous HFD-induced obesity study in mice (Eisinger et al., 2014; Fluhr et al., 2021). The results of the FPG tests for the mid-dose group provided evidence for a reduction in the glucose levels, and the total and abdominal fat mass. Acetylglycine may ameliorate fat mass accumulation and further improve glucose metabolism since the decreased capacity for adipocyte differentiation and angiogenesis is reported to alleviate lipogenesis and lipolysis activities as well as insulin resistance (Patel and Abate, 2013). Elevated acetylglycine levels have been associated with decreased risk of impaired fasting glucose and onset of diabetes (Menni et al., 2013) and improved glucose tolerance (Fluhr et al., 2021). However, this evidence may not be applicable to all situations, such as high variations in glucose reduction in our high-dose group. Further investigations are needed to estimate the dose-response effects of acetylglycine on glucose.

Our study has several strengths. We had precise measures of BF mass from DXA scans to distinguish the association of metabolites with obesity. Our study systematically examined the BF-metabolites associations and partially validated them in two independent samples with different sex and ethnicities. We confirmed our novel finding with *in vivo* experiments showing that acetylglycine can significantly affect adiposity. One inherent limitation is the difficulty harmonizing metabolites from different metabolomics platforms. Although we partially replicated our findings in other sex and ethnicity groups, more research on obesity is still needed since metabolic responses affect or are affected by diverse lifestyles and diets. Second, we cannot draw causality in our findings due to the cross-sectional design. However, our validations from the independent replication cohort and *in vivo* mice studies may support the robustness of our findings to some extent.

Our investigation of adiposity phenotypes systematically identified BF metabolomic signatures and several relevant pathways. Our study provides evidence that acetylglycine, creatinine, and cortisone may have a protective role against body fat accumulation. External validation replicated six metabolites associated with BF in either Caucasian women or African American and Caucasian men. The protective effects of acetylglycine were consistent across different samples and were further validated *in vivo* in mice. The findings open new possibilities for utilizing acetylglycine as a potential diagnostic biomarker and therapeutic target of obesity or obesity-related diseases.

## Data Availability

The data that support the findings of this study are available from the corresponding author upon request and approval of the team and respective institutions. The Kyoto Encyclopedia of Genes and Genomes database and network documents for the topological pathway analysis have been deposited on the Mendeley data website: https://data.mendeley.com/datasets/rhm2b8hz75
