# Supplementary material for: Systematic metabolomic studies identified adult adiposity biomarkers with acetylglycine associated with fat loss in vivo
Source: Front Mol Biosci. 2023 Apr 14;10:1166333. doi: 10.3389/fmolb.2023.1166333 (PMC10141311; doi:10.3389/fmolb.2023.1166333)
Supplement: Supplementary file 2 [file DataSheet1.docx]

Supplementary Material

Systematic metabolomic studies identified adult adiposity biomarkers with acetylglycine associated with fat loss *in vivo*

Kuan-Jui Su^1,2†^, Xing-Ying Chen^3,4†^, Rui Gong^1,3,5^, Qi Zhao^6^, Shi-Di Hu^4^, Mei-Chen Feng^4^, Ye Li^3^, Xu Lin^1,3,4^, Yin-Hua Zhang^3^, Jonathan Greenbaum^1^, Qing Tian^1^, Hui Shen^1^, Hong-Mei Xiao^7^, Jie Shen^3,4*^, Hong-Wen Deng^1*^

**^†^** Kuan-Jui Su and Xing-Ying Chen share first authorship.

*** Correspondence:**Hong-Wen Deng, Ph.D.: [hdeng2@tulane.edu](mailto:hdeng2@tulane.edu); Jie Shen, M.D., Ph.D: sjiesy@smu.edu.cn

# Supplemental Data

## Supplemental Information 1: Summary of the study populations

### Discovery cohort

A total of 517 unrelated peri-/post-menopausal Chinese women were recruited from the Third Affiliated Hospital of Southern Medical University (Guang Zhou, Guang Dong Province, China) from June 2014 to January 2018. The study recruitment was initially designed for our ongoing projects on metagenomic and metabolomic analysis in osteoporosis. A detailed description of the inclusion and exclusion criteria is available elsewhere (Gong et al., 2021). Briefly, the inclusion criteria for study subjects were: 1) 40 years of age or older, 2) in peri-/post-menopausal phase (irregular menstrual periods, < 12 months since final menstrual period, or no menstrual periods > 12 months) (Harlow et al., 2012), and 3) having lived in Guang Zhou City for at least three months. Exclusion criteria that could bias results include the following:

1) Diagnosis of any diseases including chronic renal disease (serum creatinine > 1.9 mg/dL), diabetes mellitus, chronic liver disease, significant chronic lung disease, rheumatoid arthritis, celiac disease, post-gastrectomy, Crohn's disease, ulcerative colitis, and any other diseases that would affect metabolic consequences

2) Alcohol use disorder, defined as having a problem controlling alcohol use and being preoccupied with alcohol at least once a week

3) Receiving medication in the past three months including antibiotics, estrogens, anticonvulsant or proton pump inhibitors

4) Underwent surgery/therapy, such as hysterectomy/bilateral ovariectomy, anticonvulsant therapy, and corticosteroid therapy at pharmacologic levels currently, or for more than 6 months duration at any time

Signed consent forms were obtained from all the participants. The clinical measurements and procedures in this study were carried out following the principles of the Helsinki Declaration II and regulations of the Third Affiliated Hospital of Southern Medical University Institutional Review Board.

### Validation cohorts

The Louisiana Osteoporosis Study (LOS) is a prospective cohort with ongoing recruitment (recruitment of a total of ~20,000 subjects targeted, ~17,000 subjects recruited as of 2022) since 2011, which aims to investigate genetic and nongenetic determinants of osteoporosis and other musculoskeletal diseases/traits (Du et al., 2017). Two independent samples from LOS were used to validate the obesity-related biomarkers. Briefly, the first validation cohort consisted of 136 Caucasian women (aged 20–40 years) from a discordant phenotype design based on the top/bottom 20% of the hip bone mineral density Z-score in the LOS cohort (Zhao et al., 2018), and the second sample consisted of 700 men (295 African Americans and 405 Caucasians aged 20–51 years). The exclusion criterion included preexisting chronic conditions relevant to bone mass as below. The studies were approved by the Tulane University Institutional Review Board.

Individuals must meet the following inclusion criteria to be eligible to participate in the study: 1) ≥ 18 years of age (to ensure peak bone mass is attained), 2) Caucasian, African American, or Asian, 3) willing to participate in the study and attend exam for bone densitometry and blood draw; and 4) can speak and understand spoken English.

We will adopt the following exclusion criteria to minimize nongenetic influence on bone mass variation so as to empirically enhance the importance of individual genetic factors for bone mass. These criteria will be assessed based on the subject’s answers.

1. Female subjects who are or could be pregnant;
2. Female subjects who have had bilateral oophorectomy;
3. Serious residuals from cerebral vascular disease;
4. Diabetes mellitus, except for those controlled under medication;
5. Chronic renal failure;
6. Chronic liver failure;
7. Significant chronic lung disease;
8. Alcohol abuse as defined by those who cannot limit drinking, get drunk regularly, and cannot fulfill major responsibilities at work, school, or home;
9. Chronic obstructive pulmonary disease (COPD);
10. Corticosteroid therapy at pharmacologic levels for more than 6 months duration;
11. Treatment with anticonvulsant therapy for more than 6 months duration;
12. Evidence of other metabolic or inherited bone disease such as hyper- or hypoparathyroidism, Paget's disease, osteomalacia, osteogenesis imperfecta or others;
13. Rheumatoid arthritis (except for minor cases that involve only hand joint and wrist);
14. Collagen disease (i.e., osteogenesis imperfecta and hypochondrogenesis);
15. Chronic gastrointestinal diseases including celiac disease, post gastrectomy, Crohn’s disease, ulcerative colitis, liver transplant, cirrhosis;
16. Upper or lower limb loss or disability;
17. HIV infection.

Since our dual-energy X-ray absorptiometry (DXA) machine cannot operate if the subject’s body weight is over 300 pounds, we will not recruit a subject whose body weight is over 300 pounds.

## Clinical measurements

A comprehensive questionnaire for all respondents in the discovery and validation cohorts was conducted by interviewer-assisted data collection to acquire information related to demographics, lifestyle (i.e., smoking, drinking, and physical activity), dietary factors, and reproductive and medical history. Trained research staff members collected clinical measurements with participants dressed in light indoor clothing without shoes: weight (kg) was measured using a calibrated balance beam scale, height (cm) was measured using a calibrated stadiometer, and body mass index was calculated as kg/m^2^. The classification of body mass index (BMI) for the Asian discovery cohort was categorized according to the cut-off points of <18.5 for underweight, 18.5–23 for normal, 23–27.5 for overweight, and >27.5 for obese (Corazon Barba, Metro Manila, Tommaso Cavalli-Sforza, Jeffery Cutter, Ian Darnton-Hill, Mabel Deurenberg-Yap, Tim Gill, Philip James, Gary Ko, Alice Ho, Shiriki Kumanyika, Anura Kurpad, Nick Mascie-Taylor, Hyun Kyung Moon, Fumio Nakadomo, Chizuru Nishida, 2004). In the validation cohorts, BMI was categorized as <18.5 for underweight, 18.5–25 for normal, 25–30 for overweight, and ≥30 for obese (About Adult BMI | Healthy Weight, Nutrition, and Physical Activity | CDC, n.d.). Physical activity was assessed using the overall hours of exercise per week in the discovery cohort and using the average number of times of exercise per week in the validation cohorts.

Daily calibrated DXA machines (discovery cohort: Lunar, GE Healthcare, Madison, WI, USA; validation cohorts: Hologic Inc., Bedford, MA, USA) were used to precisely quantify total body composition and fat content, including android and gynoid regional results. The android region of interest (ROI) was defined as lower boundary at pelvis cut, upper boundary above pelvis cut by 20% of the distance between pelvis and neck cuts, and lateral boundaries were the arm cuts. The gynoid ROI was defined as upper boundary below the pelvis cut line by 1.5 times the height of the android ROI, gynoid ROI height equal to 2 times the height of the android ROI, and lateral boundaries were the outer leg cuts. Figure S2 shows a sample scan, including android and gynoid ROI. The BF distribution phenotypes considered in this study included android fat/whole body fat mass ratio (A/W ratio), gynoid fat/whole body fat mass ratio (G/W ratio), android fat to gynoid fat ratio (A/G ratio), and whole body fat percentage (W%) (Wiklund et al., 2008; Bogl et al., 2016).

## Supplemental Information 2: Metabolomics procedure

### Metabolomics profiling in the discovery cohort.

Untargeted liquid chromatography-mass spectrometry (LC-MS) metabolite profiling was conducted on serum samples from the discovery cohort via a high-resolution tandem mass spectrometer TripleTOF5600plus (SCIEX, UK) at Lian-Chuan Biotechnology Co., Ltd. (Hangzhou, China). A high-resolution platform was used to detect metabolites eluted from the column. The quadrupole-time of flight (Q-TOF) was operated in both positive and negative ion modes for the analysis of a wide range of non-volatile metabolites. The curtain gas was set 30 pounds per square inch (PSI), Ion source gas^1^ and gas^2^ were set 60 PSI, and an interface heater temperature was 650 ℃. The range of IonSpray Voltage Floating was set at 5000 volts (V) for positive ion mode and -4500V for negative ion mode. The mass spectrometry data were acquired in information dependent acquisition mode. The time-of-flight mass range was from 60 to 1200 Dalton. The survey scans were acquired in 150 milliseconds and as many as 12 product ion scans were collected if exceeding a threshold of 100 counts per second and with a 1+ charge-state. Total cycle time was fixed to 0.56 seconds. Four-time bins were summed for each scan at a pulser frequency value of 11 kHz through monitoring of the 40 GHz multichannel time-to-digital converter with four-anode/channel detection. Dynamic exclusion was set for four seconds. During the acquisition, the mass accuracy was calibrated every 20 samples. Furthermore, in order to evaluate the stability of the LC-MS during the whole acquisition, a quality control sample (Pool of all samples) was acquired after every ten samples. The acquired MS data pretreatments including peak picking, peak grouping, retention time correction, and second peak grouping was performed using XCMS software (Tautenhahn et al., 2012). CAMERA Bioconductor package were used to annotate identified features with related isotopic peaks and adducts (Kuhl et al., 2012). Each ion was identified by its retention time and mass-to-charge ratio and converted to an electrical signal, the intensity of which is a measure of abundance of distinct ions. As whole, LC-MS data are three-dimensional matrix including each ion represented by three coordinates: retention time, mass-to-charge ratio, and intensity.

### Metabolite identification and preprocessing

To maximize the number of annotated metabolites, we considered two different confidence levels of metabolite annotation according to the guidelines of the Metabolomics Standards Initiative (<https://github.com/MSI-Metabolomics-Standards-Initiative/CIMR>). Metabolite identification and data processing for both positive and negative ion modes were performed using the R package metaX (Wen et al., 2017). For the higher confidence level 1 identification (MS2), we matched at least two orthogonal pieces of information (retention time, mass-to-charge ratio, and/or intensity) to the authentic standard by searching an in-house fragment spectrum library. The lower confidence level 2 annotation (MS1) was defined by conducting a mass-based search with a molecular weight tolerance of 10-ppm using Kyoto Encyclopedia of Genes and Genomes (KEGG) (Kanehisa and Goto, 2000) and the Human Metabolome Database (HMDB) (Wishart et al., 2007). Due to single molecular feature from untargeted mass spectrometry could be assigned to multiple metabolites, we excluded molecular features with more than one metabolite annotation to increase confidence level of MS1 metabolite annotation. The features in the quality control (QC) samples with >50% missing values and the features in the observations with >80% missing values were removed. The remaining missing values were imputed by the K-nearest neighbors method. Probabilistic quotient normalization was applied to minimize technical artifacts, and robust spline correction was carried out for the post-acquisition correction of batch effects. In addition, the relative standard deviations of the metabolic features were calculated across all samples, and those >30% were removed. All metabolites profiling were log-transformed and auto-scaled prior to the subsequent analyses.

### Metabolomics profiling in LOS male cohorts (validations).

Metabolite profiling was performed at Metabolon, Inc. (Durham, NC, USA). Received samples were immediately stored and maintained at -80^o^C. Samples were processed to precipitate proteins with methanol under vigorous shaking for 2 min (Glen Mills GenoGrinder 2000), followed by centrifugation to recover chemically diverse metabolites by using the automated MicroLab STAR® system (Hamilton Company). The extracts were then subjected to a Waters Acquity ultra-performance liquid chromatography and a Thermo Scientific Q-Exactive high resolution/accurate mass spectrometer (MS) interfaced with a heated electrospray ionization (HESI-II) source and Orbitrap mass analyzer operated at 35,000 mass resolution for positive and negative ion mode of electrospray ionization. Chromatographic separation followed by full scan mass spectra was carried out to record all detectable ions presented in the samples. The instrument and process variability were determined by the median relative standard deviation with the acceptance criteria of 6% for internal standards and 10% for the endogenous biochemicals. A series of strict quality control measures included a pooled matrix sample from a small volume of each experimental sample or from well-characterized human plasma served as a technical replicate throughout the data set, extracted water samples served as process blanks, and a cocktail of quality control (QC) standards that were chosen not to interfere with the measurement of endogenous compounds was spiked into every analyzed sample, allowing instrument performance monitoring and aiding chromatographic alignment.

Metabolon’s in-house software was used to extract raw data, identify metabolite peak with quantification using area-under-the curve, and perform the QC process. Compounds were annotated by using internal libraries (more than 3,300 commercially available purified standard compounds) based on the compound’s retention time, retention index, mass to charge ratio, and chromatographic data on all compounds in the library. The tolerance of mass match was +/- 10 ppm. The MS/MS scores were used for forward and reverse comparison between the authentic standards and observations.

## Supplemental Information 3: *In vivo* validation for acetylglycine

A total of 61 female C57BL/6J mice were purchased from the Laboratory Animal Center of Southern Medical University (Guangzhou, China) and housed in a climate-control room (20°C-25°C) with standard chow and water during five weeks of acclimation. Once mice were acclimated, they were randomized into five groups, including 1) standard chow diet with vehicle (control; n =12), 2) high fat (60% kcal from fat) diet with vehicle (HFD; n =12), 3) HFD with low-dosage acetylglycine (HFD+ACE500; 500 mg/kg; n =12), 4) HFD with medium-dosage acetylglycine (HFD+ ACE1000; 1000 mg/kg; n =12), and 5) HFD with high-dosage acetylglycine (HFD+ ACE1500; 1500 mg/kg; n =13). Two mice in the high-dosage group died and were excluded from the study. The acetylglycine dosages were determined based on a previous systematic toxicity study and modified to fit our mice model (Harper et al., 2010). Food was exchanged and weighed every four days for each cage. We administered drinking water or acetylglycine (treatment) to the corresponding mice by oral gavage administrations every day starting at eight weeks of age. The experimental measurements were recorded weekly throughout the experiments, including food consumption and body weight every week during the 16-week intervention period. In order to evaluate the effects of acetylglycine on fat distribution, we used micro-computed tomography (µ-CT) (LaTheta LCT-200; Hitachi Aloka Medical Ltd., Tokyo, Japan) to measure the total abdominal fat, visceral body fat, and subcutaneous body fat in the 14^th^ week. The procedure has been described elsewhere (Luu et al., 2009; Lee et al., 2021). In brief, the mice were maintained under anesthesia and placed in the prone position with both legs fully extended on the bed. The abdominal fat of mice was defined as the region of interest (ROI) between the L1 and L5 vertebrae (Luu et al., 2009). The abdominal visceral fat mass and subcutaneous fat mass volumes were automatically separated by the abdominal muscular wall. Finally, the volume of adipose tissue and lean tissue were converted to weight by assuming the density of 0.92 g/cm^3^ and 1.06 g/cm^3^. At the end of the experiment, mice were anesthetized and sacrificed after 12 hours of fasting.

### Biochemical analysis

Fasting plasma glucose tests were performed on a random sample of six mice from each group after six hours of fasting on Day 81, and for all the study mice after 12 hours of fasting on Day 121. Blood was collected from the lateral tail vein, and glucose levels were measured immediately using a digital glucometer (Onetouch Ultraeasy, Johnson & Johnson, America). At the end of the experiment, blood was obtained immediately via retro-orbital blood collection, left at room temperature for at least 30 minutes to obtain clotted blood samples, then centrifuged at low speed (3000×g, 10min, 4°C) to obtain serum. The serum samples were stored at -80°C and sent to Wuhan Servicebio Technology Co., Ltd. (Wuhan, China) for biochemical analysis. The levels of total cholesterol (TC), triglyceride (TG), high-density lipoprotein cholesterol (HDL), low-density lipoprotein cholesterol (LDL), alanine transaminase (ALT), and aspartate aminotransferase (AST) in the serum samples were determined by Automatic Biochemical Analyzer (Chemray 240 and Chemray 800, Rayto Life and Analytical Sciences Co., Ltd., China) following the instructions of the corresponding reagent kit.

## Supplemental Information 4: Comprehensive procedure of statistical and bioinformatics analyses

### Statistical and bioinformatics analyses in the discovery cohort

All analyses in this study were conducted under the R environment version 3.6. The sample characteristics were compared using the Kruskal-Wallis Rank Sum Test for overall test across three samples, t-test for continuous variables, and the $\chi^{2}$ test for discontinuous variables between two groups. The p-value for the characteristic comparisons is considered statistically significant with a threshold *p*-value $\leq0.05.$ To investigate the relationships between metabolites and BF traits, we performed a joint analysis for each metabolite and multivariate BF phenotypes using the seemingly unrelated regression (SUR) equations approach. The model assumes that the error terms are independent across observations but may have cross-equation correlations within observations. The SUR method adjusts the inferences for the covariance structure between the multiple equations (Henningsen and Hamann, 2007). The joint null hypothesis is that none of the traits are associated with the tested metabolite, and rejecting the null indicates that at least one trait is associated with the metabolite. The R package systemfit was used to apply the SUR equations model (Henningsen and Hamann, 2007), adjusting for BF-related covariates such as age, exercise time, annual family income, and height. The joint null hypothesis was tested using an F statistic, and we adjusted for multiple testing using the Benjamini–Hochberg false discovery rate. An FDR *q*-value $\leq$ 0.2 was considered statistically significant (Zhao et al., 2018; Bellissimo et al., 2019).

### Sparse partial least squares regression (sPLS)

To further prioritize the importance of metabolites for BF phenotypes, we applied the sparse partial least squares (sPLS) regression model, a multivariate approach which combines aspects of principal components analysis (PCA) and linear regression to maximize the covariance between the projection of metabolites and continuous outcomes. PLS is widely used in metabolomics studies due to the collinearity between functionally related metabolites. The details of the sPLS regression have been described elsewhere (Lê Cao et al., 2008). Briefly, the sPLS regression model consists of two essential components. The first is a supervised PLS method for handling various variable distributions and multicollinearity issues, while the second is a least absolute shrinkage and selection operator (LASSO) penalization combined with singular value decomposition (SVD) technique incorporated in the PLS modeling. In this study, we assigned the scaled metabolomics features as the independent matrix (X) and the four adjusted body fat (BF) traits as the dependent matrix (Y). The R package mixOmics was used to implement the sPLS model (Rohart et al., 2017). The PLS2 model with a regression mode was used to fit a linear relationship between multiple traits in Y and multiple metabolite predictors in X. The 10-fold cross-validation technique was used to tune the sPLS model for 100 iterations or a convergence threshold less than 10^-6^. The mean squared error of predictions, r-squared, and q-squared values were averaged across all folds to obtain the best-fitted model consisting of two sPLS components as our final model. We computed variable importance in projection (VIP) scores, which summarize the importance of each metabolite to the model and are calculated as a weighted sum of the squared correlations between the sPLS components and the original variables. Any metabolites with a VIP score >1 in the first sPLS component were determined to be important for at least one of the BF phenotypes.

The selection of cutoffs for the FDR and VIP score usually depends on the study purpose. In this study, we aimed to provide an adequate and comparative list of BF-related metabolites from these two multivariate methods for future replication. In addition, the metabolites identified by both methods may be prioritized for further investigation. The most important BF-related metabolites were prioritized as those with VIP score >1 and SUR FDR *q*-value $\leq$0.2. A Venn diagram was constructed using the R package RAM (Chen et al., 2018) to compare and contrast the relationships of BF-related metabolites for each phenotype.

### Topological pathway analysis

To explore vital metabolic processes and reveal potential biological mechanisms for the BF-related metabolites, a heterogeneous network approach (a knowledge-based network method) was conducted using Kyoto Encyclopedia of Genes and Genomes database (KEGG) annotated compounds to determine potential candidates of BF-related biological entities including reactions, enzymes, modules, and pathways for further analysis (Picart-Armada et al., 2017). The heat diffusion algorithm was used to quantify the propagation of flow in a given KEGG crosstalk pathway network to reflect biological perturbations of metabolites (Picart-Armada et al., 2017).

The heat diffusion process was comprehensively introduced elsewhere (Picart-Armada et al., 2018). Briefly, the heat diffusion method is defined as below and proceeded via the FELLA package:

1. Establish an undirected topological network based on the KEGG database.
   1. The reference pathway database was built using the KEGG release 90.0+/06-05, Jun 19 2019, the Homo sapiens KEGG Genes Database included 327 pathways, 117 modules, 1,135 enzymes, 5,427 reactions, and 3,964 KEGG compounds. The database for the FELLA pathway analysis is available on Mendeley Data (<http://dx.doi.org/10.17632/rhm2b8hz75.1>; DOI: 10.17632/rhm2b8hz75.1).
2. Defined the conductance matrix $KI=L+B$, where L is denoted as the unnormalized graph Laplacian matrix and B is the diagonal adjacency matrix with $B_{ii}=1$ if node I is a pathway and $B_{ii}=0$ otherwise. $i$ is a node of the given network. Then the heat equation as below to apply to the given meshed network.

$$T=-KI^{-1}\cdot G=R_{HD}\cdot G$$

Where $T$ is a stationary matrix with constant parameters, $R_{HD}$ is the linear mapping matrix to compute the temperatures, and G denotes the heat generator vector, which given a vector of metabolites contains one for identified metabolites and 0 otherwise.

1. Normalize the raw diffusion scores to reduce the potential biases caused by the network topology. The null model ($T_{null}$) is defined as below to calculate the first and second moments:

Mean$(\mu)$: $\mathbb{E}\left( T_{null} \right)=R_{HD}\mathbb{\cdot E}\left( X \right)$

Where $\mathbb{E}\left( X \right)=p*X$, p is a Bernoulli distribution and defined as $\frac{n_{in}}{n_{comp}}$, the number of infected metabolites ($n_{in})$ out of the total compounds ($n_{comp})$. $X$ is the random variable obtained by permuting $G$.

The covariance matrix$(\sigma)$: $\sum\left( T_{null} \right)=R_{HD}\cdot\sum\left( X \right)\cdot R_{HD}^{T}$

Where:

$\sum\left( X \right)=p(1-p)\cdot\left[ \begin{matrix} \begin{matrix} 1 & \rho\\ \rho& 1 \end{matrix} & \begin{matrix} \cdots\\ \cdots\end{matrix} & \begin{matrix} \rho\\ \rho\end{matrix} \\ \begin{matrix} \vdots& \vdots\end{matrix} & \ddots& \vdots\\ \begin{matrix} \rho& \rho\end{matrix} & \cdots& 1 \end{matrix} \right]$ and $\rho=-\frac{1}{n_{comp}-1}$

Then normalized diffusion score and p-scores for each node are calculated:

$z=\frac{T-\mu}{\sigma}$ and $ps=1-\Phi(z)$

Where $ps$ is the p-score of a metabolite and $\Phi$ is the cumulative standard Gaussian distribution function of z-scores. We used the default p-score less or equal as the threshold to 0.05 to rank and report the significant pathways.

All KEGG biological entities as nodes were assigned a score, reflecting their importance relative to a set of input metabolites. Following the previous definition, the diffusion score for each node was normalized to Z-scores and converted to *p*-scores for reducing the potential biases leaded by the given topological network (Picart-Armada et al., 2018), as defined:

$$ps_{i}=1-\Phi(z_{i})$$

Where $ps_{i}$ is the *p*-score of a metabolite $i$, $z_{i}$ is its z-score, and $\Phi$ is the cumulative distribution function of the standard Gaussian distribution. The *p*-score offered a probabilistic evaluation and prioritization of the pathway associations with a set of given metabolites. We considered biological components with a *p*-score < 0.05 to be significant pathways for BF-related metabolites. The R package “FELLA” was used to conduct these pathway analyses and provide the BF-related biological entities (Picart-Armada et al., 2018).

Cytoscape was used to analyze the topological properties of the identified biological components from the KEGG knowledge-based network (Shannon et al., 2003). The topological properties used to quantify the importance of biological entities included connectivity degree, betweenness centrality, and closeness centrality (Doncheva et al., 2012). The degree connectivity of each node is defined as the number of connections linked to the node, and the nodes with high degree connectivity are considered to be hub nodes. The betweenness centrality of each node reflects the amount of influence over the flow of connection on other nodes in the network. The closeness centrality is a measure of how information/influence efficiently spreads from a given node to other reachable nodes in the network. Lastly, the combined score was calculated as a sum of both betweenness and closeness scores to rank their importance in the identified network.

### Statistical analysis in validation cohorts

The metabolites of both validation cohorts were harmonized to the BF-related metabolites in the discovery cohort with the consistent Human Metabolome Database (HMDB) ID or KEGG ID, or PubChem compound ID (Kim et al., 2016). They were subjected to further validation via two association analyses: Spearman’s rank correlation test and partial Spearman’s rank correlation test between each trait and the identified metabolites for each cohort and ethnicity. Partial Spearman correlation coefficients were adjusted for age, height, physical activity for both cohorts, and ethnicity for the second overall validation cohort. The corAndPvalue function in the R package WGCNA (version 1.69) and pcor.test function in the R package ppcor (version 1.1) were used to perform Spearman’s and partial Spearman’s rank correlation tests, respectively (Langfelder and Horvath, 2008; Kim, 2015). *P*-value ≤ 0.05 was considered statistically significant in the validation cohorts.

### Statistical analysis for the *in vivo* experiment

The Generalized Estimating Equations (GEE) model was applied to investigate the associations between acetylglycine and repeated measurements for weight and weight gain. The model used an autoregressive working correlation structure, which was determined based on comparing the correlation information criterion (CIC) between different models. The covariates included baseline weight, treatment (water, low-, medium-, or high-dose acetylglycine oral gavage), and diet (normal/HFD) in the weight model, and treatment and diet in the weight gain model. The R package geepack was used to perform the analyses (Halekoh et al., 2006). Post-hoc tests for weight and weight gain comparisons were performed using the Tukey method with a significance level of 0.05. The Kruskal-Wallis test was used for the global test of significant differences between treatment groups, and the Wilcoxon test was performed for the post-hoc tests with a significance level of FDR *q*-value $\leq$ 0.05 for the effect of acetylglycine on one-time measurements (µ-CT and biochemical measurements).

## Supplemental Information 5 Data summary of annotation level in the discussion section

We chose metabolites to discuss by following one of the two criteria, which are well described in the method section. Briefly, criterion one limits the metabolites that associate with all BF traits in the discovery cohort, and criterion two selects the ones with the association replicated in validation cohorts. There are overall eight metabolites that met either of the criteria after de-duplication. Below we summarized the numbers of annotated metabolites by criterion and MS level:

Table S17 The overview of the metabolites included in the discussion section.

| Annotation level | Count (metabolite name) | | Counts of unique metabolites |
| --- | --- | --- | --- |
|  | Criterion 1 | Criterion 2 |  |
| MS2 level | 3  (Acetylglycine, Cortisone, Creatinine) | 5  (Acetylglycine, Cortisone, Creatinine, Glycocholic acid,  N-Phenylacetyl- L-Glutamine) | 5 |
| MS1 level | 2  (Pyridinoline, Deoxycoformycin) | 1  (Thiamine) | 3 |

Under criterion one, we identified three BF-associated metabolites with MS2 annotations, and further discussed them in our revised manuscript. Two MS1-level metabolites, pyridinoline and deoxycoformycin, also meet the first criteria. While they are lacking in evidence of their relationships with body fat in human metabolomics studies and biological mechanisms investigation, the associations were robust. Therefore, we consider they are potentially significant and definitely merit future research.

Our second criteria – replicated BF associations in validation cohorts – helps to limit the false identifications of BF-related metabolites. We only used high confidence compounds in both validation cohorts to provide more confident and reproducible study findings. Following this criterion, we had one metabolite, thiamine, with MS1-level annotations and had the replicated associations with body fat in female and male validations cohorts. To the best of our knowledge, our study provided the first evidence of the relationship between thiamine and body fat in *in vivo* human metabolomics studies.

To emphasize, we discussed five BF-related metabolites that are with MS2 level annotations. They are acetylglycine, cortisone, creatinine, N-Phenylacetyl- L-Glutamine, and Glycocholic acid. Three metabolites with MS1 level annotations, including thiamine, pyridinoline, and deoxycoformycin, are carefully selected by excluding the metabolic features with multiple annotations to reduce the uncertainty. Moreover, thiamine was validated in the men’s and women’s replication samples, and “pyridinoline and deoxycoformycin” were associated with all BF traits in our discovery cohort. This will improve the confidence in our findings and limit us from delivering any wrong discussion or conclusion to readers.

## Abbreviations

A/G ratio: android fat mass/gynoid fat mass ratio; A/W ratio: android fat mass/whole body fat mass ratio; AA: African American; ACE: acetylglycine; BF: body fat; BMI: body mass index; Cau: Caucasian; CIC: correlation information criterion; FDR: false discovery rate; G/W ratio: gynoid fat mass/whole body fat mass ratio; GEE: generalized estimating equation; HFD: high fat diet; MIS: Metabolomics Standards Initiative; KEGG: Kyoto encyclopedia of genes and genomes; S: subcutaneous fat mass; SD: standard deviation; SUR: seemingly unrelated regression; µ-CT: micro-computed tomography; V: visceral fat mass; V/S ratio: viscera/subcutaneous fat mass ratio; VIP: variable importance in projection score; W%: Whole body fat percentage.

# Supplementary Figures and Tables

## Supplementary Tables

**Table S1:** Characteristics of study samples.

| Sample Characteristic | **Discovery** | **Validation 1** | **Validation 2** | | |
| --- | --- | --- | --- | --- | --- |
|  | Mean (SD) or N (%) | Mean (SD) or N (%) | Mean (SD) or N (%) | | |
| Ethnicity | Han | Cau | Both | AA | Cau |
| N | 517 | 136 | 700 | 295 | 405 |
| Sex | Female | Female | Male | Male | Male |
| Age, year | 52.84 (2.92) | 31.49 (5.08) | 37.78 (8.45) | 39.06 (7.96) | 36.84 (8.68) |
| Weight, kg | 57.28 (7.74) | 70.33 (21.37) | 83.35 (16.83) | 83.25 (17.82) | 83.41 (16.09) |
| Height, cm | 157.87 (5.06) | 164.57 (6.38) | 174.91 (7.01) | 175.03 (7.24) | 174.82 (6.84) |
| BMI, kg/m^2^ | 22.97 (2.87) | 25.95 (7.53) | 27.23 (5.25) | 27.13 (5.32) | 27.31 (5.21) |
| BMI categories^#^ |  |  |  |  |  |
| Underweight | 20 (3.87 %) | 3 (2.21%) | 4 (0.57%) | 2 (0.68%) | 2 (0.49%) |
| Normal | 265 (51.26%) | 82 (60.29%) | 268 (38.29%) | 112 (37.98%) | 156 (38.52%) |
| Overweight | 193 (37.33%) | 24 (17.65%) | 234 (33.43%) | 98 (33.22%) | 142 (35.06%) |
| Obese | 39 (7.54%) | 27 (19.85%) | 194 (27.71%) | 83 (28.14%) | 105 (25.93%) |
| Annual household income categories |  |  |  |  |  |
| <36,000 Yuan | 139 (26.9%) | -- | -- | -- | -- |
| 36,000 - 120,000 Yuan | 221 (42.8%) | -- | -- | -- | -- |
| > 123,000 Yuan | 157 (30.4%) | -- | -- | -- | -- |
| Exercise time, hours/week; counts/week^*^ | 4.75 (5.33) | 3.07 (2.20)^*^ | 3.33 (3.00)^*^ | 2.82 (2.51)^*^ | 3.71 (3.27)^*^ |
| Whole body fat % | 34.23 (5.34) | 33.94 (7.18) | 23.05 (5.90) | 21.64 (5.73) | 24.07 (5.81) |
| Android fat mass, kg | 1.93 (0.61) | 1.81 (1.30) | 1.70 (1.11) | 1.50 (1.02) | 1.85 (1.15) |
| Gynoid fat mass, kg | 3.66 (0.92) | 4.74 (2.03) | 3.38 (1.43) | 3.16 (1.47) | 3.54 (1.37) |
| Android/Gynoid fat ratio | 0.53 (0.13) | 0.36 (0.13) | 0.47 (0.14) | 0.44 (0.13) | 0.49 (0.15) |
| Android fat/whole body fat ratio | 0.10 (0.01) | 0.07 (0.02) | 0.08 (0.02) | 0.07 (0.02) | 0.08 (0.02) |
| Gynoid fat/whole body fat ratio | 0.19 (0.02) | 0.20 (0.02) | 0.17 (0.02) | 0.17 (0.02) | 0.17 (0.02) |

Mean values for continuous variables are presented as mean (SD) and categorical variables are presented as a count and its percentage (%). # BMI categories for Han: Underweight: BMI < 18.5 kg/m^2^; Normal: 18.5 - 23 kg/m^2^; Overweight: 23 – 27.5 kg/m^2^; Obese: BMI ≥ 27.5; BMI categories for the validations: Underweight: BMI < 18.5 kg/m^2^; Normal: 18.5 – 25 kg/m^2^; Overweight: 25 – 30 kg/m^2^; Obese: BMI ≥ 30; *: Exercise time for the validations: count/per week. SD, standard deviation; BMI, body mass index; Cau: Caucasian; AA: African American

**Table S2:** Sample characterization and comparison between samples

| Sample Characteristic  Mean (SD) or N (%) | Discovery (D) | Validation 1 (V1) | Validation 2 (V2) | *P*-value^*^ | | | | |
| --- | --- | --- | --- | --- | --- | --- | --- | --- |
|  |  |  |  | Overall^a^ | D vs V1 | D vs V2 | V1 vs V2 | V2:  AA vs Cau |
| N | 517 | 136 | 700 | -- | -- | -- | -- | -- |
| Age, year | 52.84 (2.92) | 31.49  (5.08) | 37.78  (8.43) | <0.001 | <0.001 | <0.001 | <0.001 | 0.001 |
| Weight, kg | 57.28 (7.74) | 70.34 (21.37) | 83.35  (16.83) | <0.001 | <0.001 | <0.001 | <0.001 | **ns** |
| Height, cm | 157.87 (5.06) | 164.57 (6.38) | 174.91  (7.01) | <0.001 | <0.001 | <0.001 | <0.001 | **ns** |
| BMI, kg/m2 | 22.97 (2.87) | 25.96  (7.53) | 27.23  (5.25) | <0.001 | <0.001 | <0.001 | 0.017 | **ns** |
| Exercise time, hours/week; counts/week* | 4.75 (5.33) | 3.07  (2.20) | 3.33  (3.00) | NA | NA | NA | **ns** | <0.001 |
| Whole body fat % | 34.23 (5.39) | 33.94  (7.18) | 23.05  (5.90) | <0.001 | **ns** | <0.001 | <0.001 | <0.001 |
| Android fat mass, kg | 1.93 (0.61) | 1.81  (1.30) | 1.70  (1.11) | <0.001 | 0.125 | <0.001 | **ns** | <0.001 |
| Gynoid fat mass, kg | 3.66 (0.92) | 4.74  (2.03) | 3.38  (1.43) | <0.001 | <0.001 | <0.001 | <0.001 | <0.001 |
| Android/Gynoid fat ratio | 0.53 (0.13) | 0.36  (0.13) | 0.47  (0.14) | <0.001 | <0.001 | <0.001 | <0.001 | <0.001 |
| Android fat/whole body fat ratio | 0.10 (0.01) | 0.07  (0.02) | 0.08  (0.02) | <0.001 | <0.001 | <0.001 | <0.001 | <0.001 |
| Gynoid fat/whole body fat ratio | 0.19 (0.02) | 0.20  (0.02) | 0.17  (0.02) | <0.001 | 0.001 | <0.001 | <0.001 | 0.013 |

a) Characteristics of study subjects compared across three groups using Kruskal-Wallis Rank Sum Test. * Characteristics of study subjects compared between the study cohorts and race using t-test for continuous variables and χ2 test for discontinuous variables. p ≥0.05 is considered statistically significant. SD: standard deviation; NA: not available; ns: not statistically significant; Cau: Caucasian; AA: African American.

**Table S3:** Measured metabolite features in discovery samples.

| Ion mode | All feature | All annotated | MS^2^ | MS^1^ KEGG | MS^1^ HMDB |
| --- | --- | --- | --- | --- | --- |
| pos | 3202 | 1685 | 219 | 1028 | 1507 |
| neg | 2674 | 1390 | 162 | 859 | 1056 |
| Total | 5876 | 3075 | 381 | 1887 | 2563 |

Number of metabolite peaks identified in each ion mode and passed quality control in the untargeted LC-MS metabolomics platform. Note: Positive or negative ion switching to analyze serum metabolites. QC, Quality control; KEGG, Kyoto Encyclopedia of Genes and Genomes database; HMDB, Human metabolome database.

**Table S4:** Metabolites significantly associated with body fat traits in the discovery cohorts.

| Class | Metabolite | m/z | RT | *P*-value | | | | |
| --- | --- | --- | --- | --- | --- | --- | --- | --- |
|  |  |  |  | Joint^*^ | A/G | A/W | G/W | W |
| -- | Giganin | 596.4912 | 41.59 | **3.61**$\boldsymbol{\times1}\boldsymbol{0}^{\boldsymbol{-4}}$ | **1.46**$\boldsymbol{\times1}\boldsymbol{0}^{\boldsymbol{-2}}$ | 2.07$\times10^{-1}$ | **2.94**$\boldsymbol{\times1}\boldsymbol{0}^{\boldsymbol{-4}}$ | **1.53**$\boldsymbol{\times1}\boldsymbol{0}^{\boldsymbol{-4}}$ |
| Alkaloids and derivatives | Cytochalasin Ppho | 510.2904 | 29.42 | **8.92**$\boldsymbol{\times1}\boldsymbol{0}^{\boldsymbol{-4}}$ | **7.35**$\boldsymbol{\times1}\boldsymbol{0}^{\boldsymbol{-3}}$ | 1.05$\times10^{-1}$ | **1.44**$\boldsymbol{\times1}\boldsymbol{0}^{\boldsymbol{-4}}$ | **1.79**$\boldsymbol{\times1}\boldsymbol{0}^{\boldsymbol{-3}}$ |
| Benzenoids | 1,2-Benzenedicarboxylic acid | 187.005 | 24.26 | **8.55**$\boldsymbol{\times1}\boldsymbol{0}^{\boldsymbol{-4}}$ | **6.48**$\boldsymbol{\times1}\boldsymbol{0}^{\boldsymbol{-3}}$ | 1.44$\times10^{-1}$ | **2.65**$\boldsymbol{\times1}\boldsymbol{0}^{\boldsymbol{-3}}$ | **2.65**$\boldsymbol{\times1}\boldsymbol{0}^{\boldsymbol{-3}}$ |
|  | Sulfadimethoxine | 348.077 | 214.65 | **1.51**$\boldsymbol{\times1}\boldsymbol{0}^{\boldsymbol{-3}}$ | 1.26$\times10^{-1}$ | 9.96$\times10^{-1}$ | **7.21**$\boldsymbol{\times1}\boldsymbol{0}^{\boldsymbol{-3}}$ | **1.49**$\boldsymbol{\times1}\boldsymbol{0}^{\boldsymbol{-3}}$ |
| Carcinogens | Tris(2-chloroethyl)phosphate | 282.9452 | 372.21 | **1.84**$\boldsymbol{\times1}\boldsymbol{0}^{\boldsymbol{-3}}$ | **2.45**$\boldsymbol{\times1}\boldsymbol{0}^{\boldsymbol{-2}}$ | 7.91$\times10^{-2}$ | **9.73**$\boldsymbol{\times1}\boldsymbol{0}^{\boldsymbol{-4}}$ | **3.81**$\boldsymbol{\times1}\boldsymbol{0}^{\boldsymbol{-2}}$ |
| Lipids | Glycocholic acid | 464.3094 | 190.57 | **6.77**$\boldsymbol{\times1}\boldsymbol{0}^{\boldsymbol{-4}}$ | **5.60**$\boldsymbol{\times1}\boldsymbol{0}^{\boldsymbol{-3}}$ | 1.72$\times10^{-1}$ | **4.64**$\boldsymbol{\times1}\boldsymbol{0}^{\boldsymbol{-5}}$ | **2.30**$\boldsymbol{\times1}\boldsymbol{0}^{\boldsymbol{-2}}$ |
|  | Cucurbitacin C | 559.3298 | 180.98 | **1.07**$\boldsymbol{\times1}\boldsymbol{0}^{\boldsymbol{-3}}$ | 1.32$\times10^{-1}$ | 7.65$\times10^{-1}$ | **1.58**$\boldsymbol{\times1}\boldsymbol{0}^{\boldsymbol{-3}}$ | **2.38**$\boldsymbol{\times1}\boldsymbol{0}^{\boldsymbol{-3}}$ |
|  | 28-Homobrassinolide | 510.3819 | 41.76 | **1.73**$\boldsymbol{\times1}\boldsymbol{0}^{\boldsymbol{-3}}$ | **3.67**$\boldsymbol{\times1}\boldsymbol{0}^{\boldsymbol{-2}}$ | 5.69$\times10^{-1}$ | **3.79**$\boldsymbol{\times1}\boldsymbol{0}^{\boldsymbol{-4}}$ | 5.72$\times10^{-2}$ |
|  | D-Glucosyldihydrosphingosine | 479.3679 | 40.71 | **2.18**$\boldsymbol{\times1}\boldsymbol{0}^{\boldsymbol{-3}}$ | **1.48**$\boldsymbol{\times1}\boldsymbol{0}^{\boldsymbol{-2}}$ | 1.86$\times10^{-1}$ | **3.80**$\boldsymbol{\times1}\boldsymbol{0}^{\boldsymbol{-4}}$ | **2.56**$\boldsymbol{\times1}\boldsymbol{0}^{\boldsymbol{-3}}$ |
|  | Cortisone^+^ | 361.2 | 36.25 | **2.21**$\boldsymbol{\times1}\boldsymbol{0}^{\boldsymbol{-3}}$ | **4.43**$\boldsymbol{\times1}\boldsymbol{0}^{\boldsymbol{-3}}$ | **5.49**$\boldsymbol{\times1}\boldsymbol{0}^{\boldsymbol{-3}}$ | **5.76**$\boldsymbol{\times1}\boldsymbol{0}^{\boldsymbol{-3}}$ | **1.88**$\boldsymbol{\times1}\boldsymbol{0}^{\boldsymbol{-3}}$ |
| Organic acids | Austalide J | 443.2057 | 29.29 | **2.50**$\boldsymbol{\times1}\boldsymbol{0}^{\boldsymbol{-7}}$ | **4.00**$\boldsymbol{\times1}\boldsymbol{0}^{\boldsymbol{-7}}$ | **5.75**$\boldsymbol{\times1}\boldsymbol{0}^{\boldsymbol{-4}}$ | **3.69**$\boldsymbol{\times1}\boldsymbol{0}^{\boldsymbol{-9}}$ | 7.28$\times10^{-2}$ |
|  | Pyridinoline | 444.2085 | 29.34 | **8.12**$\boldsymbol{\times1}\boldsymbol{0}^{\boldsymbol{-7}}$ | **3.07**$\boldsymbol{\times1}\boldsymbol{0}^{\boldsymbol{-6}}$ | **1.70**$\boldsymbol{\times1}\boldsymbol{0}^{\boldsymbol{-3}}$ | **1.40**$\boldsymbol{\times1}\boldsymbol{0}^{\boldsymbol{-8}}$ | **3.24**$\boldsymbol{\times1}\boldsymbol{0}^{\boldsymbol{-3}}$ |
|  | Creatinine | 112.0501 | 168.21 | **1.33**$\boldsymbol{\times1}\boldsymbol{0}^{\boldsymbol{-4}}$ | **1.01**$\boldsymbol{\times1}\boldsymbol{0}^{\boldsymbol{-5}}$ | **1.54**$\boldsymbol{\times1}\boldsymbol{0}^{\boldsymbol{-4}}$ | **2.36**$\boldsymbol{\times1}\boldsymbol{0}^{\boldsymbol{-4}}$ | **4.61**$\boldsymbol{\times1}\boldsymbol{0}^{\boldsymbol{-2}}$ |
|  | Acetylglycine | 116.0338 | 263.35 | **2.40**$\boldsymbol{\times1}\boldsymbol{0}^{\boldsymbol{-4}}$ | **1.09**$\boldsymbol{\times1}\boldsymbol{0}^{\boldsymbol{-5}}$ | **6.22**$\boldsymbol{\times1}\boldsymbol{0}^{\boldsymbol{-5}}$ | **4.34**$\boldsymbol{\times1}\boldsymbol{0}^{\boldsymbol{-5}}$ | **3.97**$\boldsymbol{\times1}\boldsymbol{0}^{\boldsymbol{-2}}$ |
|  | Alpha-N-phenylacetyl-L-glutamine | 263.101 | 214.4 | **4.27**$\boldsymbol{\times1}\boldsymbol{0}^{\boldsymbol{-4}}$ | **1.77**$\boldsymbol{\times1}\boldsymbol{0}^{\boldsymbol{-2}}$ | 4.07$\times10^{-1}$ | **1.18**$\boldsymbol{\times1}\boldsymbol{0}^{\boldsymbol{-3}}$ | **9.22**$\boldsymbol{\times1}\boldsymbol{0}^{\boldsymbol{-4}}$ |
| Organic oxygen compounds | Gemcitabine diphosphate | 421.993 | 26.19 | **2.86**$\boldsymbol{\times1}\boldsymbol{0}^{\boldsymbol{-4}}$ | **3.57**$\boldsymbol{\times1}\boldsymbol{0}^{\boldsymbol{-2}}$ | 3.25$\times10^{-1}$ | **7.47**$\boldsymbol{\times1}\boldsymbol{0}^{\boldsymbol{-3}}$ | **4.03**$\boldsymbol{\times1}\boldsymbol{0}^{\boldsymbol{-5}}$ |
| Organoheterocyclic compounds | Thiamine^+^ | 288.103 | 212.74 | **2.28**$\boldsymbol{\times1}\boldsymbol{0}^{\boldsymbol{-4}}$ | 5.64$\times10^{-2}$ | 6.63$\times10^{-1}$ | **2.77**$\boldsymbol{\times1}\boldsymbol{0}^{\boldsymbol{-3}}$ | **8.54**$\boldsymbol{\times1}\boldsymbol{0}^{\boldsymbol{-5}}$ |
|  | Deoxycoformycin^+^ | 269.1239 | 301.44 | **6.65**$\boldsymbol{\times1}\boldsymbol{0}^{\boldsymbol{-4}}$ | **1.41**$\boldsymbol{\times1}\boldsymbol{0}^{\boldsymbol{-4}}$ | **9.99**$\boldsymbol{\times1}\boldsymbol{0}^{\boldsymbol{-4}}$ | **5.14**$\boldsymbol{\times1}\boldsymbol{0}^{\boldsymbol{-5}}$ | **2.22**$\boldsymbol{\times1}\boldsymbol{0}^{\boldsymbol{-2}}$ |

*: *P*-value for overall testing; +: Positive ion mode, otherwise negative ion mode; *P*-value: Calculated via each regression model; **Bold:** *p*-value < 0.05; m/z: Mass-to-charge ratios; RT: Retention time (second); A/G: Android to gynoid ratio; A/W: Android fat to whole body total fat; G/W: Gynoid fat to whole body total fat; W: whole body total fat %

**Table S5:** Ethnicity-specific correlation analysis in the second validation cohorts.

| Metabolite | Class | Ion | m/z | RI | Molecular | Trait | $\boldsymbol{\rho}$ | $\boldsymbol{\rho}$  *p-*value | $\boldsymbol{\rho}^{'}$ | $\boldsymbol{\rho}^{'}$*p-*value |
| --- | --- | --- | --- | --- | --- | --- | --- | --- | --- | --- |
| Validation 2: African American men (n=295) | | | | | | | | | | |
| Acetylglycine | Amino acids | Polar | 116.04 | 1780 | C_4_H_7_NO_3_ | AG | -0.23 | 5.52$\times{10}^{-5}$ | -0.23 | 6.55$\times{10}^{-5}$ |
|  |  |  |  |  |  | AW | -0.27 | 1.68$\times{10}^{-6}$ | -0.26 | 5.33$\times{10}^{-6}$ |
|  |  |  |  |  |  | W% | -0.26 | 7.48$\times{10}^{-6}$ | -0.24 | 3.39$\times{10}^{-5}$ |
| Cortisone | Lipid | Neg | 359.19 | 4575 | C_21_H_28_O_5_ | AG | -0.17 | 4.22$\times{10}^{-3}$ | -0.16 | 6.52$\times{10}^{-3}$ |
|  |  |  |  |  |  | AW | -0.15 | 9.16$\times{10}^{-3}$ | -0.14 | 1.82$\times{10}^{-2}$ |
|  |  |  |  |  |  | W% | -0.16 | 7.31$\times{10}^{-3}$ | -0.15 | 1.20$\times{10}^{-2}$ |
| Glycocholic acid | Lipid | Neg | 464.30 | 5163 | C_26_H_43_NO_6_ | W% | -0.16 | 6.65$\times{10}^{-3}$ | -0.17 | 4.51$\times{10}^{-3}$ |
| N-Phenylacetyl-L-Glutamine | Peptide | Pos Early | 265.12 | 2145 | C_13_H_16_N_2_O_4_ | AW | -0.08 | ns | -0.14 | 1.72$\times{10}^{-2}$ |
|  |  |  |  |  |  | W% | -0.14 | 1.59$\times{10}^{-2}$ | -0.18 | 1.61$\times{10}^{-3}$ |
| Validation 2: Caucasian men (n=405) | | | | | | | | | | |
| Acetylglycine | Amino acids | Polar | 116.04 | 1780 | C_4_H_7_NO_3_ | AG | -0.26 | 1.06$\times{10}^{-7}$ | -0.26 | 2.03$\times{10}^{-7}$ |
|  |  |  |  |  |  | AW | -0.28 | 1.65$\times{10}^{-8}$ | -0.27 | 4.96$\times{10}^{-7}$ |
|  |  |  |  |  |  | GW | 0.15 | 2.97$\times{10}^{-3}$ | 0.14 | 3.85$\times{10}^{-3}$ |
|  |  |  |  |  |  | W% | -0.27 | 2.65$\times{10}^{-8}$ | -0.25 | 3.11$\times{10}^{-7}$ |
| Creatinine | Amino acids | Pos Early | 114.07 | 2055 | C_4_H_7_N_3_O | W% | -0.14 | 3.61$\times{10}^{-3}$ | -0.17 | 5.85$\times{10}^{-4}$ |
| Glycocholic acid | Lipid | Neg | 464.30 | 5163 | C_26_H_43_NO_6_ | WG | -0.10 | 4.63$\times{10}^{-2}$ | -0.06 | ns |
|  |  |  |  |  |  | AW | -0.11 | 2.16$\times{10}^{-2}$ | -0.08 | ns |
| N-Phenylacetyl-L-Glutamine | Peptide | Pos Early | 265.12 | 2145 | C_13_H_16_N_2_O_4_ | AG | -0.00 | ns | -0.12 | 1.94$\times{10}^{-2}$ |
|  |  |  |  |  |  | W% | -0.03 | ns | -0.10 | 4.29$\times{10}^{-2}$ |

m/z: mass-to-chare ratio; RI: Retention index; $\rho$ : Spearman correlation coefficient; $\rho^{'}$: Partial Spearman correlation coefficient adjusted age, height, and physical activity in Validation 2; ns: not statistically significant; The directions of the correlation test in the validation studies are consistent with the findings in the Chinese women cohort.

**Table S6:** Topological metabolic pathway analysis.

Excel Document: Menu_MetaboChineseObesity_SupTable.xlsx

**Table S7:** Correlation analysis in the Caucasian women.

Excel Document: Menu_MetaboChineseObesity_SupTable.xlsx

**Table S8:** Partial correlation analysis in the Caucasian women.

Excel Document: Menu_MetaboChineseObesity_SupTable.xlsx

**Table S9:** Correlation analysis in the African American and Caucasian men.

Excel Document: Menu_MetaboChineseObesity_SupTable.xlsx

**Table S10:** Partial correlation analysis in the African American and Caucasian men.

Excel Document: Menu_MetaboChineseObesity_SupTable.xlsx

**Table S11:** Correlation analysis in the African American men.

Excel Document: Menu_MetaboChineseObesity_SupTable.xlsx

**Table S12**: Correlation analysis in the Caucasian men.

Excel Document: Menu_MetaboChineseObesity_SupTable.xlsx

**Table S13:** Partial correlation analysis in the African American men.

Excel Document: Menu_MetaboChineseObesity_SupTable.xlsx

**Table S14:** Partial correlation analysis in the Caucasian men.

Excel Document: Menu_MetaboChineseObesity_SupTable.xlsx

**Table S15:** Assessment of mice abdominal fat by micro-computed tomography.

| Variable  [mean (SD)] | Overall | Stratify by Group | | | | | |
| --- | --- | --- | --- | --- | --- | --- | --- |
|  |  | Control | HFD | HFD ACE 500 | HFD ACE 1000 | HFD ACE 1500 | *P*-value |
| n | 58 | 12 | 12 | 12 | 12 | 10 | -- |
| Total Ab Fat Mass (g) | 0.65 (0.42) | 0.27 (0.12) | 1.00 (0.45) | 0.84 (0.53) | 0.56 (0.17) | 0.55 (0.15) | <0.001 |
| Visceral Fat Mass (g) | 0.47 (0.32) | 0.18 (0.09) | 0.75 (0.37) | 0.61 (0.39) | 0.39 (0.12) | 0.40 (0.11) | <0.001 |
| Subcutaneous Fat Mass (g) | 0.18 (0.10) | 0.09 (0.04) | 0.25 (0.08) | 0.23 (0.15) | 0.17 (0.05) | 0.15 (0.04) | <0.001 |
| Fat Weight Ratio | 14.71 (7.64) | 6.66 (2.53) | 22.54 (7.90) | 17.82 (7.64) | 13.61 (3.78) | 12.56 (2.90) | <0.001 |
| V/S Mass Ratio | 2.51 (0.55) | 2.05 (0.32) | 2.90 (0.74) | 2.64 (0.47) | 2.37 (0.45) | 2.61 (0.31) | 0.001 |

Total Ab Fat Mass: The total abdominal fat mass between L1–L5 of the lumbar vertebrae; HFD: High fat diet; ACE: Acetylglycine (dose); SD: Standard deviation; V/S Mass ratio: Visceral fat mass/subcutaneous fat mass; *p*-value: Overall comparison by Kruskal-Wallis Rank Sum Test

**Table S16:** Result of the biomedical analysis.

| Item  [mean (SD)] | Overall | Stratify by Group | | | | |  |
| --- | --- | --- | --- | --- | --- | --- | --- |
|  |  | Control | HFD | HFD  ACE 500 | HFD  ACE 1000 | HFD  ACE 1500 | *P*-value |
| n | 30 | 6 | 6 | 6 | 6 | 6 |  |
| FPG (mmol/L)  (6 hour fasting) | 6.62 (1.46) | 5.93 (1.16) | 8.57 (1.49) | 6.23 (0.98) | 5.68 (0.78) | 6.67 (0.90) | 0.001 |
| n | 59 | 12 | 12 | 12 | 12 | 11 |  |
| FPG (mmol/L) (12 hour fasting) | 4.55 (0.72) | 4.42 (0.54) | 4.82 (0.50) | 4.86 (0.50) | 3.86 (0.37) | 4.83 (1.07) | 0.001 |
| n | 54 | 11 | 12 | 11 | 12 | 8 |  |
| ALT (U/L) | 37.04 (17.51) | 43.32 (29.89) | 34.99 (8.81) | 32.76 (9.24) | 41.02 (19.48) | 31.40 (6.64) | 0.550 |
| AST (U/L) | 174.83 (50.25) | 174.12 (46.84) | 178.19 (29.97) | 161.47 (25.81) | 203.04 (82.03) | 146.79 (21.90) | 0.156 |
| TG (mmol/L) | 0.76 (0.40) | 0.60 (0.22) | 0.77 (0.41) | 0.88 (0.29) | 0.82 (0.36) | 0.73 (0.70) | 0.073 |
| CHO (mmol/L) | 2.75 (0.47) | 2.06 (0.18) | 2.87 (0.34) | 2.84 (0.34) | 3.02 (0.40) | 2.97 (0.22) | <0.001 |
| HDL (mmol/L) | 1.54 (0.29) | 1.18 (0.10) | 1.62 (0.24) | 1.58 (0.27) | 1.67 (0.28) | 1.66 (0.14) | <0.001 |
| LDL (mmol/L) | 0.42 (0.06) | 0.35 (0.06) | 0.42 (0.06) | 0.44 (0.04) | 0.44 (0.07) | 0.44 (0.06) | 0.006 |

CHO: Cholesterol, TG: triglyceride; HDL: High-density lipoprotein cholesterol; LDL: Low-density lipoprotein cholesterol; AST: Aspartate aminotransferase; ALT: Alanine aminotransferase; FPG: Fasting plasma glucose; HFD: High fat diet; ACE: Acetylglycine (dose); SD: standard deviation; *p*-value: Overall comparison by Kruskal-Wallis Rank Sum Test.

## Supplementary Figures


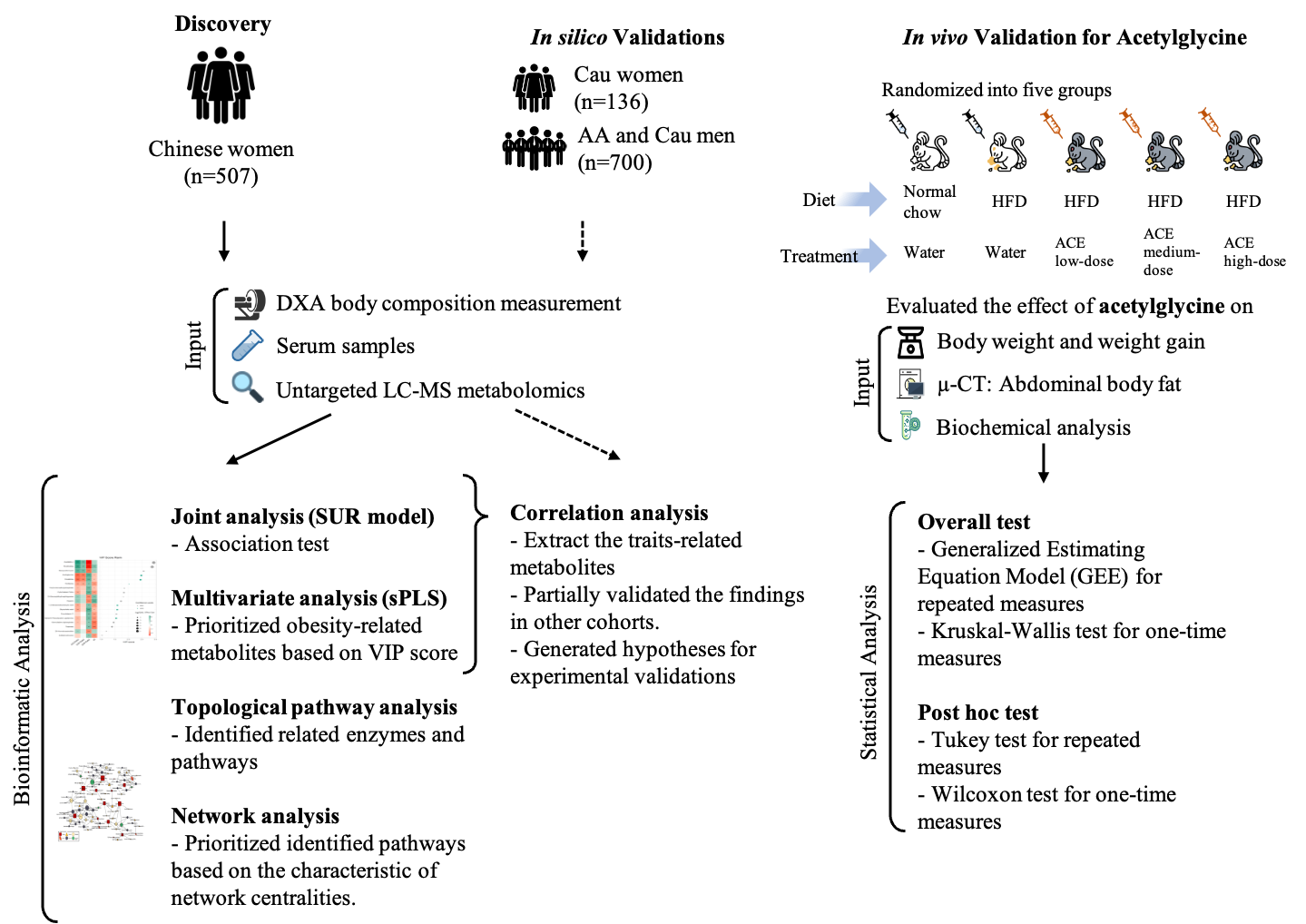


**Figure S1:** Summary of experimental design and analytical pipeline. Cau: Caucasian; AA: African American; DXA: Dual-energy X-ray absorptiometry; LC-MS: Liquid chromatography mass spectrometry; SUR: Seemingly unrelated regression; sPLS: Sparse partial least squares regression; HFD: High fat diet; ACE: Acetylglycine; µ-CT: micro-computed tomography.


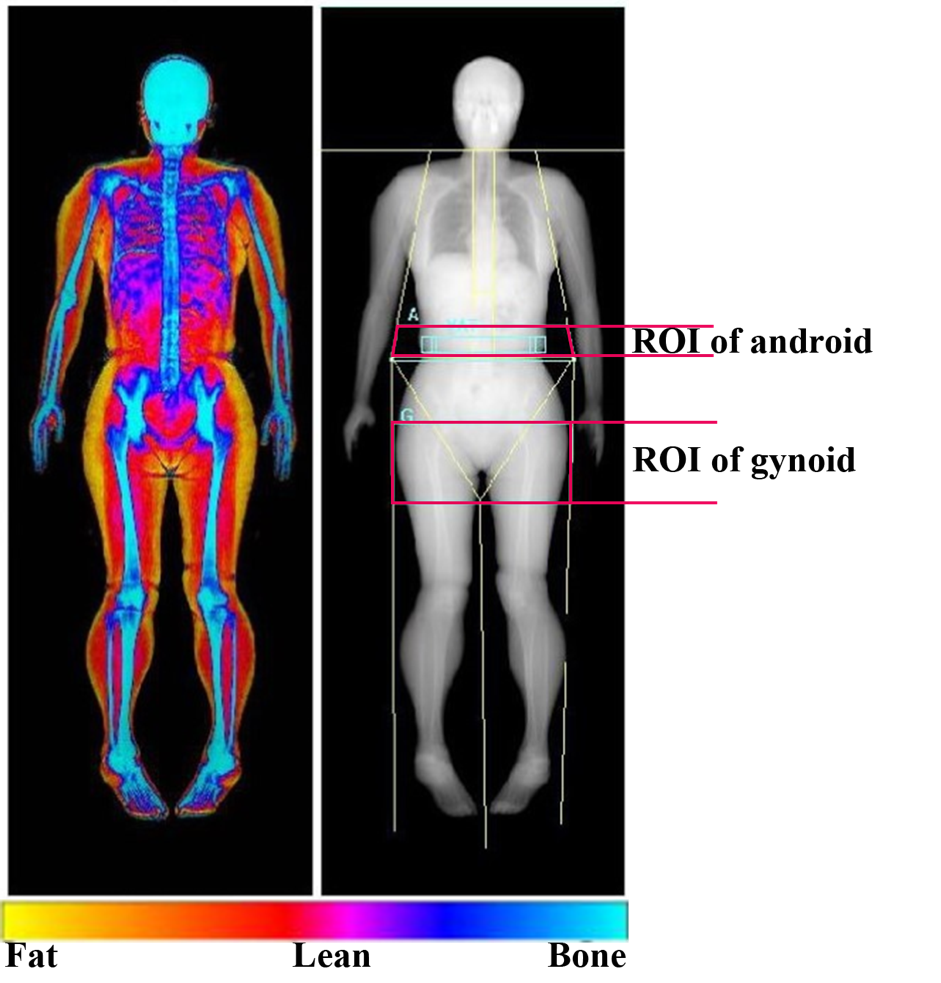


Figure S2: Measurement of regions of interest (ROI) in android and gynoid by dual-energy X-ray absorptiometry is shown in the right panel.


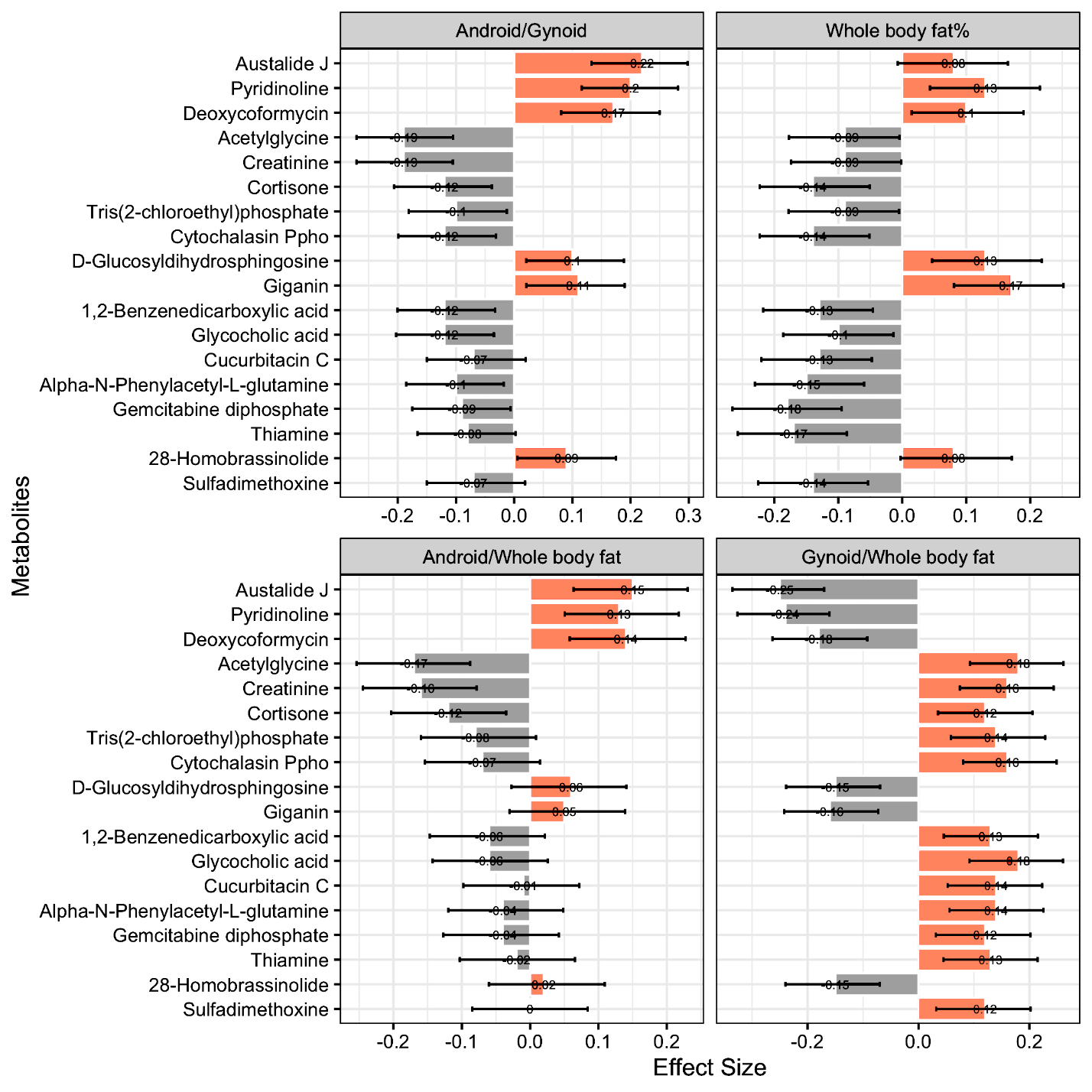


Figure S3: Effect sizes of the significant metabolites for each body fat trait. Each effect size estimates the change in the mean of each trait per standard deviation increase in the relative abundance of a metabolite. The error bars represent the 95% confidence intervals for the effect size of each metabolite. The metabolites on the y-axis were ordered by VIP score.


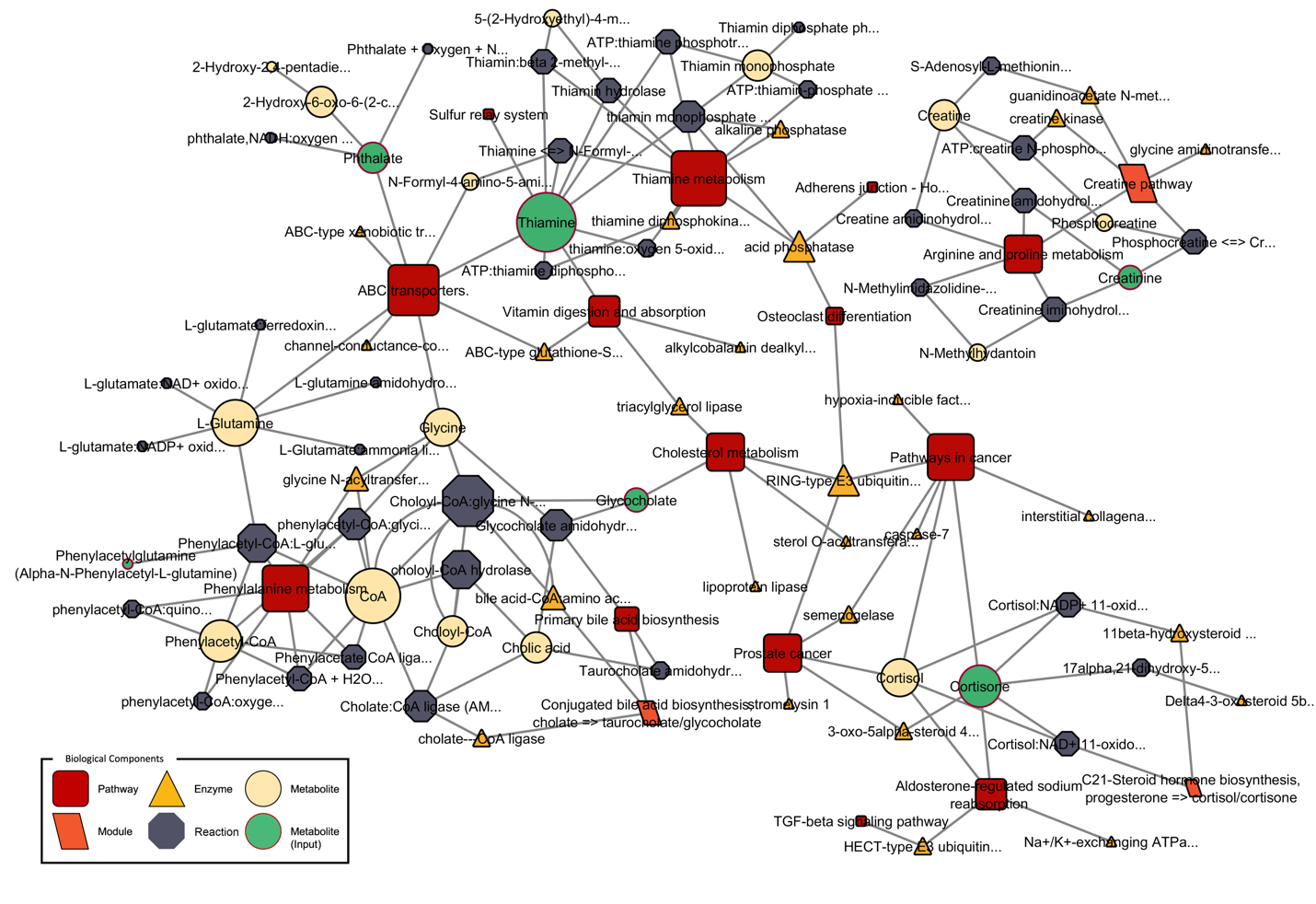


Figure S4: Result of functional topological pathway analysis by FELLA. The network reveals how the BF-related metabolites reached the relevant enzymes, reactions, and pathways and how these biological components cross talk. The size of each node was displayed according to connectivity degrees. The connections (edges) were obtained directly from KEGG annotations and displayed if *p*-value ≤0.05. The highest connectivity degree in the network was 10 from the BF-related metabolite thiamine.


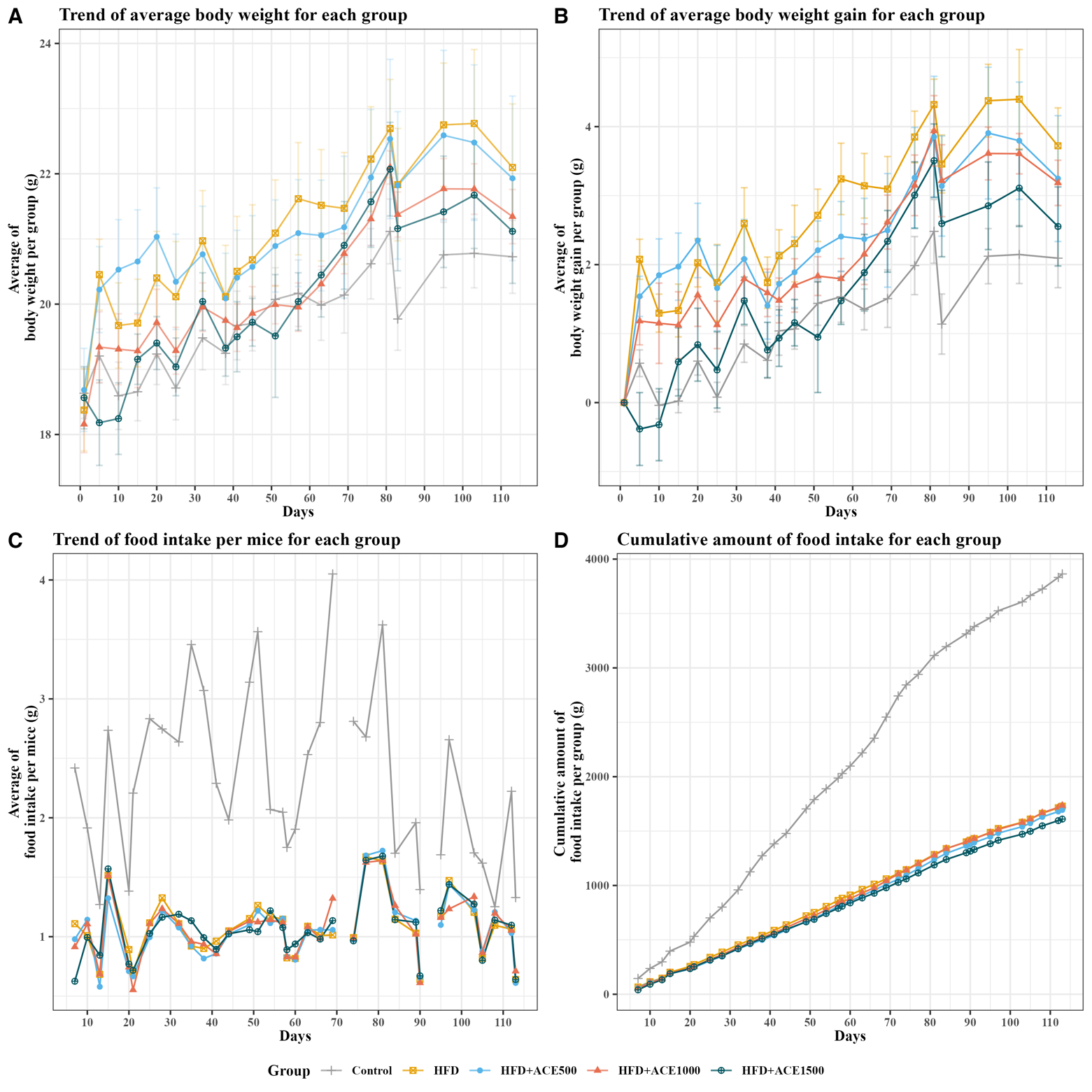


Figure S5: Body weight and food intake in mouse model of acetylglycine. (A) Trend of the mean of the development of body weight (grams) over time for each experimental group. (B) Trend of the mean of mouse body weight gain (grams) for each experimental group. (C) Average food intake per mouse in each experimental group. (D) Cumulative food consumption for each experimental group.


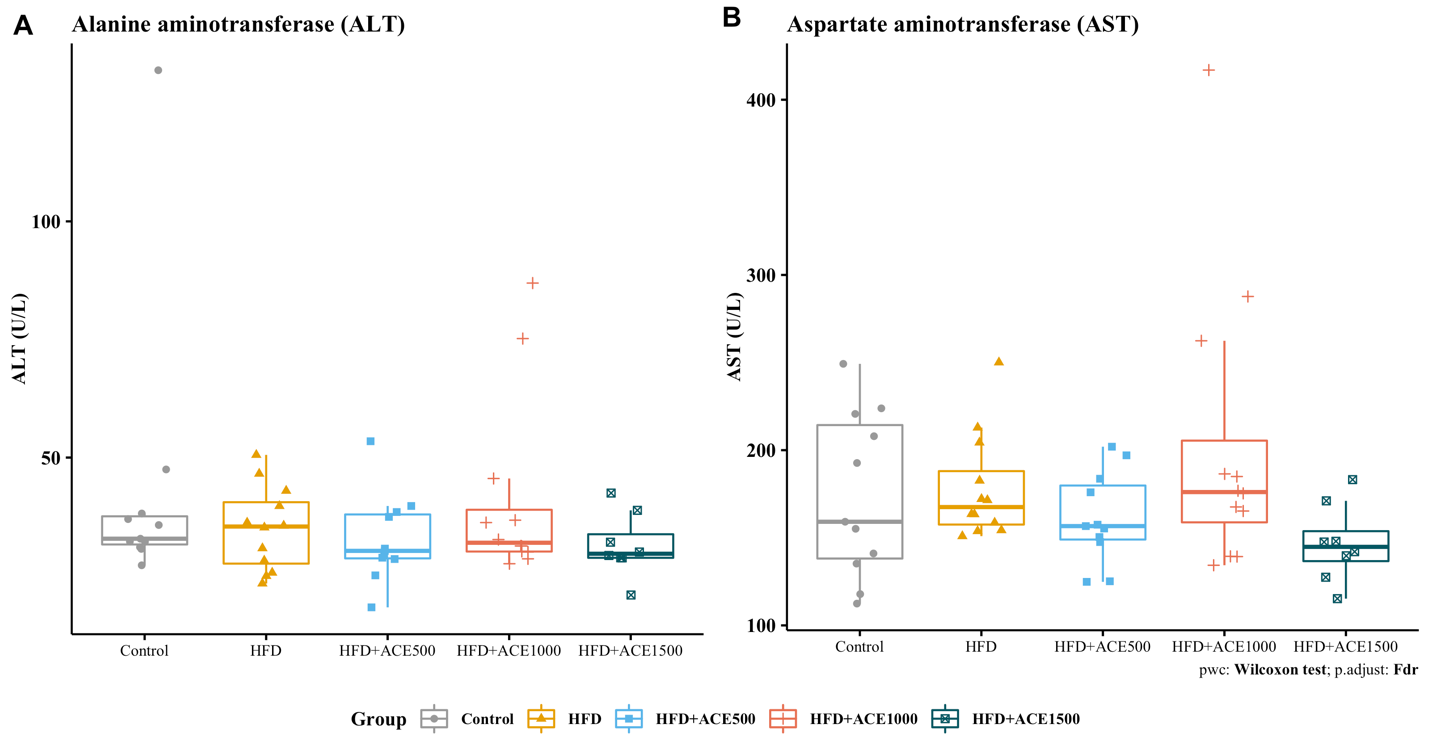


Figure S6: Box plots of measurements of liver function tests. (A) Alanine transaminase. (B) Aspartate aminotransferase. FDR: false discovery rate; *: FDR *q*-value < 0.05; **: FDR *q*-value < 0.01; ***: FDR *q*-value < 0.001; ****: FDR *q*-value < 0.0001


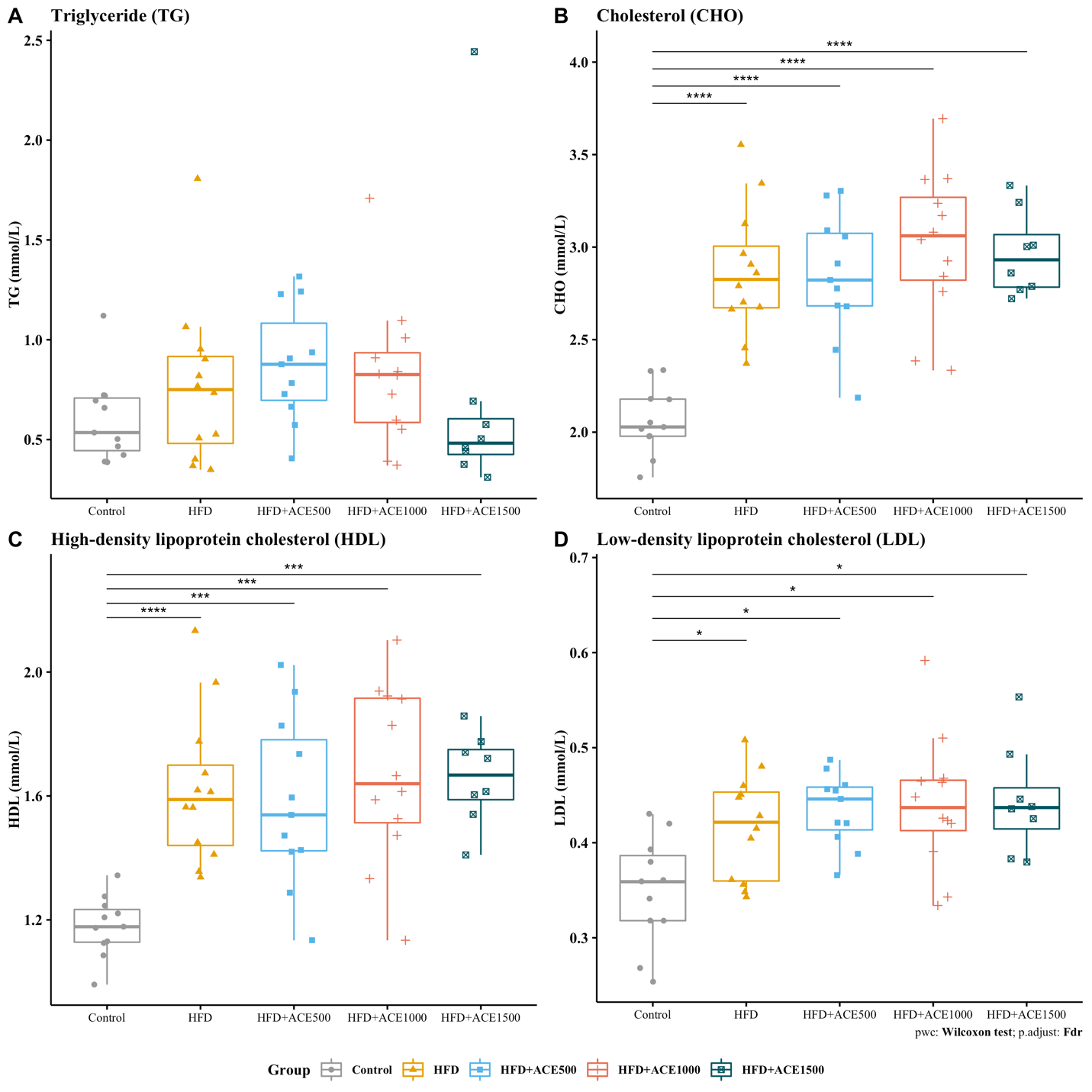


Figure S7: Box plots of measurements of cholesterol tests. (A) Triglyceride, (B) Total cholesterol, (C) High-density lipoprotein cholesterol, (D) Low-density lipoprotein cholesterol. FDR: false discovery rate; *: FDR *q*-value < 0.05; **: FDR *q*-value < 0.01; ***: FDR *q*-value < 0.001; ****: FDR *q*-value < 0.0001
